# The Effect of Glycol Side Chains on the Assembly and Microstructure of Conjugated Polymers

**DOI:** 10.1021/acsnano.2c09464

**Published:** 2022-12-14

**Authors:** Stefania Moro, Nicholas Siemons, Oscar Drury, Daniel A. Warr, Thomas A. Moriarty, Luís M. A. Perdigão, Drew Pearce, Maximilian Moser, Rawad K. Hallani, Joseph Parker, Iain McCulloch, Jarvist M. Frost, Jenny Nelson, Giovanni Costantini

**Affiliations:** † Department of Chemistry, University of Warwick, Coventry CV4 7AL, United Kingdom; ‡ School of Chemistry, University of Birmingham, Birmingham B15 2TT, United Kingdom; § Department of Physics, Imperial College London, South Kensington, London SW7 2AZ, United Kingdom; ∥ Department of Chemistry, University of Oxford, Oxford OX1 3TA, United Kingdom; ⊥ Physical Science and Engineering Division, King Abdullah University of Science and Technology (KAUST), Thuwal 23955-6900, Saudi Arabia

**Keywords:** conjugated polymers, glycolated side chains, scanning tunneling microscopy, molecular dynamics, microstructure

## Abstract

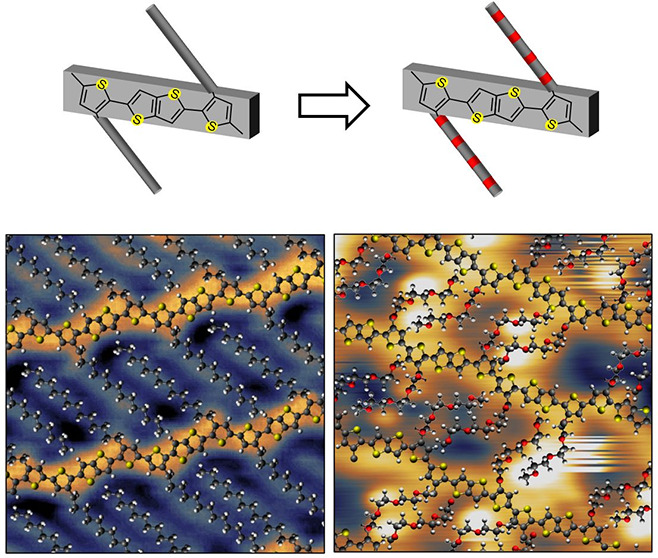

Conjugated polymers with glycol-based chains, are emerging as a material class with promising applications as organic mixed ionic-electronic conductors, particularly in bioelectronics and thermoelectrics. However, little is still known about their microstructure and the role of the side chains in determining intermolecular interactions and polymer packing. Here, we use the combination of electrospray deposition and scanning tunneling microscopy to determine the microstructure of prototypical glycolated conjugated polymers (pgBTTT and p(g2T-TT)) with submonomer resolution. Molecular dynamics simulations of the same surface-adsorbed polymers exhibit an excellent agreement with the experimental images, allowing us to extend the characterization of the polymers to the atomic scale. Our results prove that, similarly to their alkylated counterparts, glycolated polymers assemble through interdigitation of their side chains, although significant differences are found in their conformation and interaction patterns. A model is proposed that identifies the driving force for the polymer assembly in the tendency of the side chains to adopt the conformation of their free analogues, i.e., polyethylene and polyethylene glycol, for alkyl or ethylene glycol side chains, respectively. For both classes of polymers, it is also demonstrated that the backbone conformation is determined to a higher degree by the interaction between the side chains rather than by the backbone torsional potential energy. The generalization of these findings from two-dimensional (2D) monolayers to three-dimensional thin films is discussed, together with the opportunity to use this type of 2D study to gain so far inaccessible, subnm-scale information on the microstructure of conjugated polymers.

Organic π-conjugated polymers are an attractive class of materials that combine structural and electronic adaptability to deliver high charge-carrier mobility and ample possibilities of chemical tuning.^1^ The solution processability of conjugated polymers also offers inexpensive and simpler methods of device production, providing an alternative to traditional sputtering and evaporation methods for inorganic and small-molecular semiconductor materials.^2−4^ As a consequence, conjugated polymers have found use in a wide variety of optoelectronic devices, ranging from organic photovoltaic solar cells^3,5^ to organic field effect transistors (OFETs),^6,7^ organic light-emitting diodes,^8^ and organic electrochemical transistors (OECTs).^9^ In contrast to the high degree of crystallinity exhibited by small molecular organic semiconductors, the near amorphous nature of many π-conjugated polymers leads to a much greater complexity of their structure and to the frequent absence of long-range order. Despite this, it has been shown that improving short-range assembly can help in achieving highly efficient OFET devices with high charge carrier mobilities.^10,11^

Thiophene-based conjugated polymers have historically been of particular interest due to their impressive charge carrier mobility and excellent electronic properties.^12^ High hole mobility values have been observed for systems with pronounced π–π stacking, in a lamellar microstructure, allowing for an efficient charge transport to be attained.^13^ Alkyl side chains, initially incorporated into these conjugated polymers to increase their solubility,^14^ were shown to have a crucial impact on the polymer assembly and the formation of a regular microstructure.^6,7,15^ This has been extensively demonstrated for regioregular poly(3-alkylthiophene)s, in particular for the widely studied poly(3-hexylthiophene) (P3HT),^16^ where it was shown that lamellae of π-stacked backbones are stabilized by the lateral interactions of their side chains that hold together the three-dimensional (3D) structure of the material. Optimizing interactions between the side chains is, therefore, important for improving the structural order of the assembly and the charge carrier mobility of the material.^17,18^ This has led to investigations of changes in the polymer structure that can maximize the self-assembly, most notably resulting in the synthesis of poly(2,5-bis(3-alkylthiophene-2-yl)thieno[3,2-*b*]thiophene) (pBTTT, Figure 1a). pBTTT combines ideal side chain attachment density that allows for maximized interdigitation (in contrast to P3HT, where no side chain interdigitation is observed) and a largely planar backbone, as a consequence of the thienothiophene units, enabling efficient π–π stacking. Due to its highly ordered, semicrystalline structure, pBTTT shows hole mobilities >0.1 cm^2^ V^–1^ s^–1^, comparable to small molecule semiconductors and has become a benchmark in the field of conjugated polymers.^19−23^

**Figure 1:**
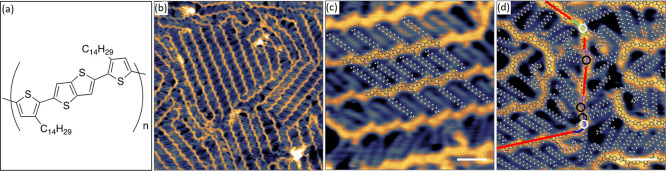
(a) Chemical structure of pBTTT-C_14_. (b) Larger scale STM image showing an extended and compact region of highly interdigitated pBTTT/Ag(111). (c) Smaller scale STM image of a highly dense region, with a scaled molecular model overlaid on part of the image. (d) Smaller-scale STM image of a relatively disorder area of pBTTT. A molecular model is overlaid, and kinks of the backbones are highlighted for one polymer strand. White circles correspond to *cis* T–T bonds, black circles correspond to *cis* T–TT bonds, while the colored lines match those of Figure S4. Scale bars are equal to 8 nm in (b) and to 2 nm in (c) and (d). Scanning parameters in constant current mode: sample bias −1.5 V, current set point 100 pA.

An emerging field of application for conjugated polymers is that of organic mixed ionic-electronic conductors, in particular for bioelectronics, with increasingly advanced devices being explored from brain-machine interfaces to “second skin” technologies.^24,25^ Specific attention has been given to the development of biosensors based on OECTs that have thin films of conjugated polymers as their active channels. This type of application requires materials capable of a combined and correlated ionic and electronic charge transport, with the former needed for directly interacting with the biotic environment and the latter for transducing the biosignal into an electronically processable one.^26,27^ Conjugated polymers with hydrophilic molecular structures are especially attractive in this regard because, besides their efficient electronic charge transport, they also show ionic conductivity, either when they already contain ionic species or through their ability to solvate ions. The best known example of the first type of such polymers is poly(3,4-ethylenedioxythiophene) doped with poly(styrenesulfonate) (PEDOT:PSS) which currently is the most used OECT channel material, partly due to its wide commercial availability. However, its main limitation is that PEDOT:PSS operates in depletion mode, while semiconducting polymers can operate in accumulation mode, with greater sensitivity and lower power consumption. Moreover, the two-component structure of PEDOT:PSS limits the possibility to engineer and optimize the material for specific applications.^28^ In fact, it is believed that the coupling of electronic and ionic charge transport in PEDOT:PSS strictly depends on its complex microstructure, which is still not fully understood and very difficult to control. Currently this represents the strongest limitation to the reliable use of PEDOT:PSS as a basis for new materials development.^29^ On the other hand, it has been reported that ion uptake can be induced by chemical design of the polymer side chains.^30^ This observation has started a new area of research based on the idea of using the backbone structure of “more traditional” OFET-conjugated polymers—already designed and optimized specifically for electronic transport, with the caveat that the HOMO energy level must be shallow enough to allow oxidation within the electrochemical window of the aqueous electrolyte—and to modify the chemical composition of their side chains in order to achieve efficient ionic transport. A significant advantage of this approach is that the chemistry of the side chains is mostly decoupled from that of the backbones, so that these two characteristics can be independently tuned. Moreover, the diffusion of external ions originating from a biosignal into the polymer thin films and in close proximity to the backbones can modify their electron and hole mobility under electrochemical bias, thus realizing the required coupling between ionic and electronic transport. For these reasons, this approach has emerged as a promising innovation in the field of conjugated polymers for biosensing.^5^ Glycols and, in particular, ethylene glycol (EG) are the simplest and most obvious candidates for substituting the alkyl side chains of traditional conjugated polymers, having historically been used as chemical interfaces with biological systems, with PEGylation being a common compatibilizing technique in pharmaceutical applications. Currently, electrochemically doped EG-functionalized materials are rivalling and even exceeding the performance of PEDOT:PSS in OECTs.^30,31^

It is however important to note that, even if the side chains do not directly influence the intrinsic charge-carrier mobility of the backbones, as mentioned above, they do play an essential role in determining the microstructure of polymer films, which in turn deeply affects the mixed conduction properties of the material and the efficiency in devices.^30^ Consequently, understanding the structural effects of exchanging alkyl for glycol side chains in conjugated polymers is of central importance in determining accurate structure–function relationships for this new class of materials. At present, only a few experimental studies based on X-ray diffraction exist, which provide valuable, but relatively limited, microscopic structural information, especially on the noncrystalline regions of the materials.^32,33^ Overall, no detailed experimental study of the impact of side chain chemistry on polymer chain interactions is currently available in the literature for this class of conjugated polymers.

Here, we aim to close this gap by directly comparing the microstructure of conjugated polymers that differ only in the nature of their alkylated and glycolated side chains. In order to keep the study at a very fundamental level and to extract general trends that go beyond the specific investigated systems, we analyze the model alkylated polythiophene polymer pBTTT and compare it with two of its glycolated analogue isomers, namely poly(2-(4,4′-bis(2-methoxyethoxy)-5′-methyl-[2,2′-bithiophen]-5-yl)-5-methylthieno[3,2-*b*]thiophene) (pgBTTT) and poly(2-(3,3′-bis(2-(2-(2-methoxy ethoxy)ethoxy)ethoxy)-[2,2′-bithiophen]-5-yl)thieno[3,2-*b*]thiophene) (p(g2T-TT)). pBTTT was chosen because, as mentioned above, it is an extensively studied prototypical model system for “traditional” conjugated polymers, assembling in a highly regular semicrystalline structure characterized by compact interdigitation of its alkyl side chains.^19,21−23^ pgBTTT and p(g2T-TT) are the simplest analogues of pBTTT since they have the same backbone, but triethylene glycol (TEG) side chains instead of alkyl ones. The only difference between them is the regioposition where the TEG chains are connected to the backbone.^9,34,35^ A direct insight into the similarities and differences of the assemblies of these polymers is gained by exploiting the high-resolution imaging capability of scanning tunneling microscopy (STM) coupled with ultrahigh vacuum (UHV) electrospray deposition (ESD), an innovative technique that has shown great potential in characterizing conjugated polymers on the submonomer scale.^6,9,36−39^ These measurements are complemented by molecular dynamics (MD) simulations of the same polymers adsorbed on a gold surface which reproduce the experiments in great detail, thereby allowing us to access the properties of the assembly all the way down to the atomic scale. Our results show that, while the glycolated analogues of pBTTT organize in less extended and regular structures, the interaction between their side chains still plays an essential role in determining both the assembly and the conformation of polymer chains. By carefully analyzing both experimental and simulation data, we demonstrate that the differences in the conformation and packing of these two types of polymers can be traced back to the different chemical nature of alkyl and EG chains and, therefore, to their different interaction motifs and energies.

## Results and Discussion

### pBTTT

Submonolayer thin films of pBTTT-C_14_^23^ (Figure 1a) were prepared by ESD, onto atomically clean and flat Au(111)/mica or Ag(111)/mica substrates in UHV and measured in situ by STM.^36,39^ Examples of the resulting images are shown in Figure 1b and Figure S2, demonstrating that the polymers form highly ordered structures extending over hundreds of nanometers. These are characterized by locally parallel backbones appearing as bright, linear structures with dimmer, straight, protruding features that correspond to the alkyl side chains. pBTTT appears to have a strong tendency to self-assemble into extended regular networks. Even in areas of lower local polymer coverage, instead of adopting a uniform surface density, pBTTT forms compact two-dimensional (2D) islands, leaving areas of bare exposed substrate (Figure S3). No loose polymer strands were observed, suggesting a strong tendency to maximize intermolecular interactions.

Higher resolution images, such as in Figure 1c, show periodic details within the backbone that are consistent with the length and shape of BTTT repeat units adsorbed with their molecular plane parallel to the surface. This is clearly demonstrated by superposing on the STM images scaled molecular models of pBTTT that have been geometry-optimized with the Avogadro molecular editor using the MMFF94 force field (see section 4 of the Supporting Information, SI). The models show an excellent fit with the zigzag appearance of the backbone that is caused by the relative orientation of the thiophene and thienothiophene repeat units, as well as with the C_14_ alkyl side chains binding to the thiophene subunits (see Figure 1c). The measured periodicity along the backbone of (13.8 ± 0.07) Å is compatible with the values in the literature.^23^Figure 1c also shows very clearly that the side chains of neighboring polymer strands are highly interdigitated. A statistical analysis of the areas of highest packing density resulted in a local average backbone separation of (19.5 ± 1.0) Å and a side chain tilt angle of about 44° ± 2° (measured from the normal to the backbone).

**Figure 2:**
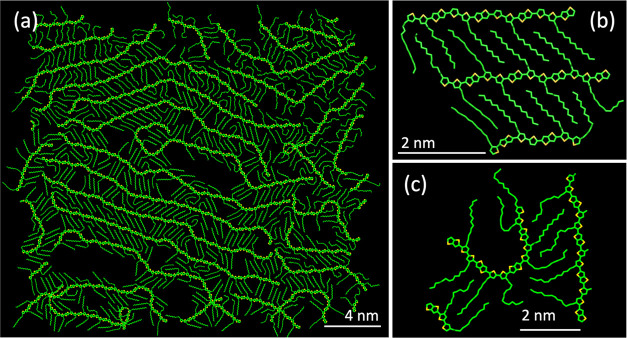
(a) Representative snapshot of MD simulations of high-density pBTTT-C_14_ deposited on a Au substrate and simulated for 5 ns. The image clearly shows pBTTT’s strong tendency to pack with interdigitated side chains and backbones lying parallel to each other. (b) Close-up image of the highest density packing regions, showing the distinct angle of 43° formed between the straight side chains and the backbones. (c) Close-up image showing a less ordered region with a bent backbone and the associated disruption in the side chain packing required to accommodate it.

In order to rationalize these experimental findings, MD simulations were performed. The polymers were represented by 20-mers, initially equilibrated in vacuum and subsequently placed 3 Å above a gold substrate (modeled by using a 12-6 van der Waals (vdW) potential^40^) where they adsorbed and further evolved for 5 ns (+ 20 ns in the case of high molecular density; see the SI for further methodological details). In all cases, their conformations were not observed to significantly change after adsorption, on the time scales analyzed by MD. Larger area snapshots of the simulated oligomers after equilibration (see, for example, Figure 2a) show a great similarity with the STM images, while providing at the same time detailed information on the atomic positions that are not directly accessible in the experimental data. The simulations confirm the face-on adsorption of the pBTTT backbones, their local parallel arrangement, the mostly straight conformation of the side chains, and the strong tendency of the polymer to aggregate into extended 2D islands. Furthermore, the simulations demonstrate that, even when starting from isolated molecules, the intermolecular interactions of pBTTT cause the polymers to assemble into a dense packing, characterized by a significant fraction of interdigitating side chains. In the highest molecular density regions, the side chains are in the all-*trans* conformation and form an average angle of 43° with respect to the backbone normal (Figure 2b), in close agreement with the values extracted from the STM images.

The type of packing revealed by ESD-STM experiments and MD simulations is analogous to that expected in 3D thin films of pBTTT, where it was shown that the microstructure of the polymer is composed of lamellae of π-stacked polymers that interact with each other by interdigitation of their side chains.^21^ Grazing incidence wide-angle X-ray scattering (GIWAXS) and atomic force microscopy (AFM) measurements both agreed in the measurement of the lamellar spacing, i.e., the 3D equivalent of the backbone separation in 2D STM images, of about 2 nm and a corresponding side chain tilt angle of about 44°.^17,22,23^ These values are strikingly similar to those discussed above, giving a first indication that the patterns of intermolecular interactions imaged by STM and simulated by MD, closely resemble those regulating the polymer assembly in functional 3D thin films.

Kline et al. also proposed a simple side chain packing model to explain the assembly of pBTTT, which is based on the optimization of the side chain interdigitation.^17^ The fundamental assumption of the model is that the ideal configuration of the alkyl side chains is the one they would adopt if they were free (as opposed to being connected to the polymer backbone), i.e., the all-trans conformation of crystalline polyethylene (PE). Due to its simplicity, this model can be generalized to predict the interdigitation geometry of a generic conjugated polymer with (linear) alkyl side chains as a function of the only relevant synthetic controllable parameter, i.e. the spacing of side chains along the backbone (or of its reciprocal, the so-called side chain attachment density).^17^ In the case of pBTTT, the model predicts that the ideal side chain packing is attained for side chains tilted by an angle close to 44°, in excellent agreement with the value obtained from the experimental measurements.^17^

Although the backbones of pBTTT are mostly straight, they do not always have a simple linear configuration and occasional more complex combinations of straight segments, kinks, and broader bends are observed (Figure 1b,d). To better understand how the conformation of the pBTTT backbones is influenced by rotations around their single C–C bonds, we performed simple geometry optimizations in Avogadro of several selected configurations of isolated pBTTT oligomers, where bonds between thiophene (T) and thienothiophene (TT) subunits were rotated by 180° with respect to each other (Figure S4). This analysis shows that the lowest energy configuration, where all T and TT units are *trans* to each other, with respect to the orientation of the sulfur atoms, produces a straight backbone and that deviations from this fully straight geometry require a certain number of T–T or T–TT bonds to be in a *cis* conformation. More precisely, any pronounced kink in the backbone must arise because of *cis* T–T bonds, while *cis* T–TT bonds do not produce as much of a distinct bend in the backbone (see SI). Varying the number and position of *cis* bonds alters the resulting kinking pattern and the position of the side chains with respect to the backbone.

Based on this information, it is possible to fit tailored molecular models onto high-resolution STM images, as those shown in Figure 1d, where the details of the non-straight polymer sections can be distinguished. By trying to carefully reproduce the different features along the backbones and, in particular, the position and orientation of the side chains, an excellent overall match can be reached. It is even possible to identify *cis* T–TT bonds which, although not producing any major kink (Figure S4biii), do change the relative position of successive side chains with respect to the backbone. A statistical analysis could thus be carried out by fitting molecular models in multiple STM images, which resulted in the fraction of T–T and T–TT bonds that were found in the *cis*, rather than *trans*, configuration to be 10% and 7%, respectively (Table S1).

Similar types of conformations and occurrences of *cis* T–T and T–TT bonds were observed in the MD simulations (Figure S13). While simulated *cis* frequencies are close to those experimentally observed, the simulations that are equilibrated in vacuum will not be able to capture effects in the experimental data that result from chain assembly in solution during ESD. The measured *cis* and *trans* frequencies could in principle be traced back to the difference in torsional potential energy between the *cis* and *trans* configurations of T–T and T–TT bonds. However, in the case of interacting polymers, kinks in the backbone also cause a local loss of side chain interdigitation (see Figures 1d and 2c), and this energetic cost is likely to be greater than the energy difference between the *trans* and *cis* configurations (typically predicted to be in the 40–60 meV energy range by density functional theory (DFT) calculations^9,41^). As a consequence, we suggest that the measured distribution of *cis* and *trans* configurations is mostly determined by the favorable enthalpic interactions between alkyl side chains in an interdigitated configuration, rather than the torsional energetics of the backbone. A similar result had already been found for the 2D monolayer assembly of conjugated polymers based on the diketopyrrolopyrrole acceptor unit.^36^

### pgBTTT and p(g2T-TT)

**Figure 3:**
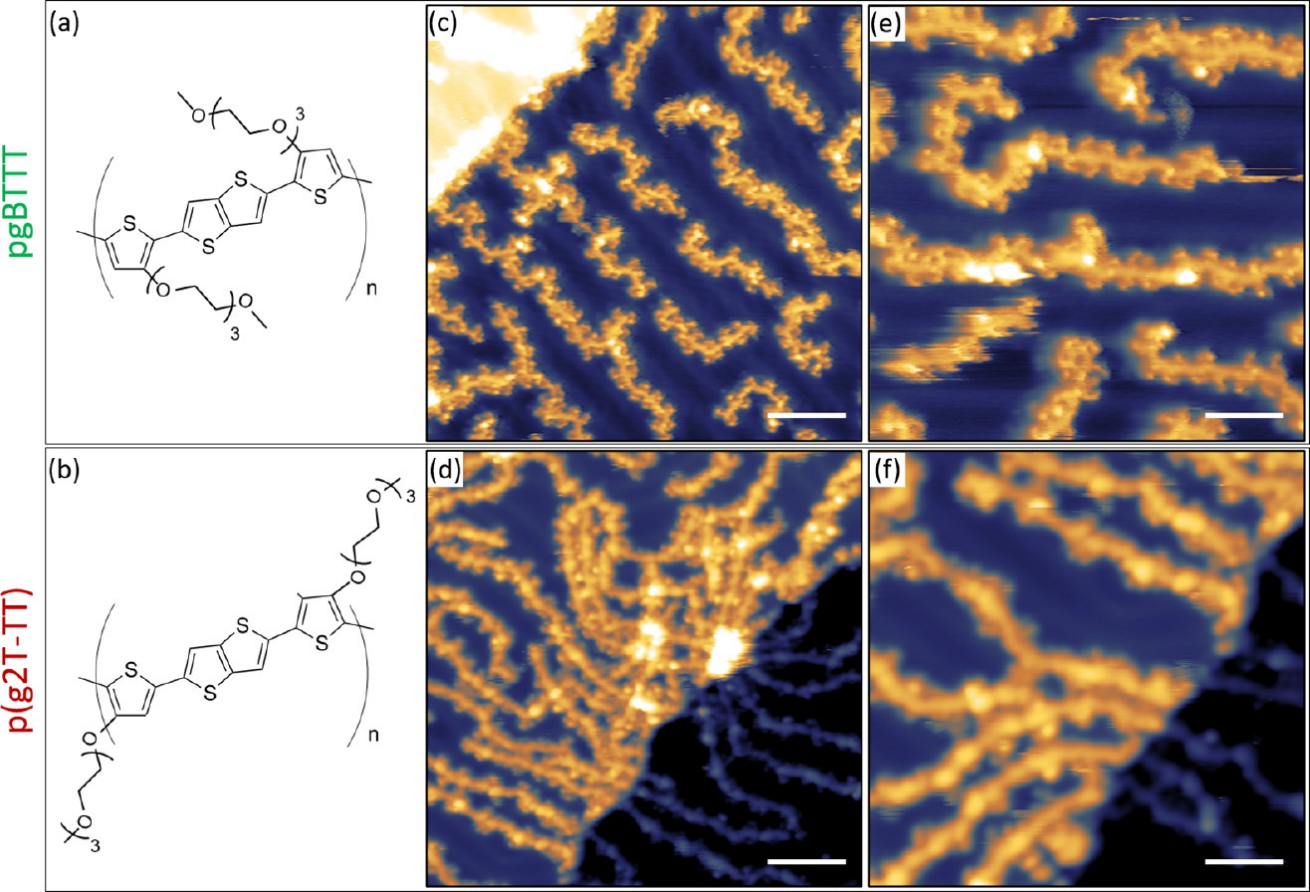
(a) and (b) Chemical structures of pgBTTT and p(g2T-TT), respectively, showing the different side chains attachment position of the two isomers. (c) and (e) STM images of low coverage areas of pgBTTT deposited in UHV by ESD on a Au(111) surface. (d) and (f) Analogous STM images for p(g2T-TT); an atomic Au(111) step is present in these images. The reluctance of the polymers to assemble at low surface coverages can be clearly seen. All images were acquired at 77 K. Scale bars in (c) and (d) correspond to 8 nm; in (e) and (f) to 5 nm. Scanning parameters in constant current mode: (c) −1.5 V, 70 pA; (d) +1.6 V, 75 pA; (e) −1.2 V, 75 pA; (f) +1.6 V, 75 pA.

The glycolated analogues of pBTTT were obtained by replacing its C_14_ alkyl side chains with triethylene glycol ones.^9,30^ This specific type of chain was chosen because earlier studies showed it to be a good compromise to obtain both high electronic and ionic charge transport, exhibiting the best efficiencies in OECT devices.^34^ The TEG chains were connected to the pBTTT backbone by attaching them to either the 3 and 3′ positions or to the 4 and 4′ positions of the thiophenes, resulting in the two isomers, pgBTTT^9^ and p(g2T-TT),^30^ whose structures are shown in Figure 3a,b, respectively. The presence of an oxygen atom in the side chain α position causes an intramolecular O···S interaction that is not present in pBTTT. The different side chain attachment positions of the two glycolated polymers produce different side chains-to-backbone interactions and, as a result, a different optimum backbone conformation.^42^

**Figure 4:**
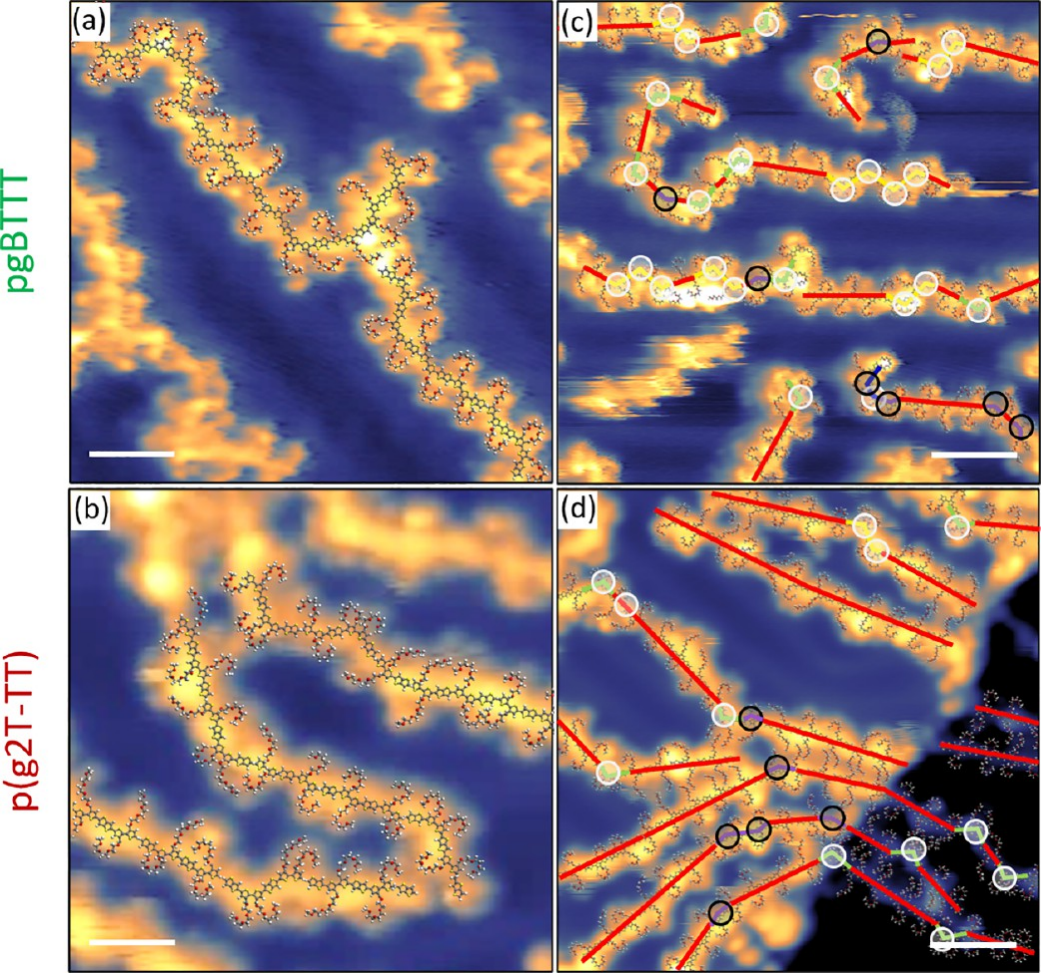
Fitting of molecular models onto STM images of low surface density pgBTTT and p(g2T-TT) absorbed polymers. (a) and (b) Small-scale images with the full molecular models including TEG side chains bent to different degrees. (c) and (d) Effect of backbone kinks on larger scale images. White circles correspond to *cis* T–T bonds, black circles correspond to *cis* T–TT bonds, while the colored lines match those of Figure S4. Scale bars are equal to 3 nm for (a) and (b) and to 5 nm in (c) and (d). Scanning parameters in constant current mode: (a) −1.5 V, 70 pA; (b) +1.6 V, 75 pA; (c) −1.2 V, 75 pA; (d) +1.6 V, 75 pA.

Both pgBTTT and p(g2T-TT) were deposited in conditions similar to those used for pBTTT onto an atomically flat and clean Au(111)/mica surface by ESD in UHV and studied in situ by STM. Contrary to their alkylated analog, these polymers exhibit much more distinct low and high surface coverage areas. At low coverages, pgBTTT and p(g2T-TT) molecules tend to spread out, seemingly avoiding the interaction with each other and maximizing their relative distance (Figure 3c–f). This behavior seems to be more pronounced in the case of pgBTTT, as p(g2T-TT) chains were still found to occasionally interact with each other even at lower coverages. In these lower polymer density regions, it was possible to acquire high-resolution images, where individual molecules could clearly be seen with bright, elongated central parts corresponding to the backbones (adsorbed face-on on the substrate) and lateral features extending from them. The periodicity of the latter matches that of the polymer repeat unit, and they can thus be identified as the TEG side chains. Interestingly, the side chains are not straight, as in the case of pBTTT, but curved to various degrees (Figure 3c–f). Rotations around C–C and C–O bonds can cause the TEG chains to adopt a conformation that deviates from being linear. Successive oxygen atoms in a TEG chain can be either anti or gauche to each other and, in the latter case, there are two different possibilities, as subsequent gauche oxygen atoms can be oriented either side of the previous one (Figure S6a). To analyze these conformations in more detail, molecular models of TEG were created, and their geometry was optimized in Avogadro. These models show that while a fully linear chain can only be obtained if all the oxygen atoms are anti with respect to each other, a higher proportion of gauche oxygens results in a more curved TEG (Figure S6b). A further important parameter in determining the extent of the bending is the type of gauche conformations, with alternating gauches producing curved chains, while a sequence of gauche rotations all in the same direction causes the TEG chain to spiral into a helix. Molecular models with TEG chains in different conformations were fit to the STM images showing that by combining anti and gauche conformations, it is possible to accurately represent the different curvatures experimentally observed for the side chains (Figure 4a,b). What also appears quite evident is that the TEG side chains exhibit a wide variation of conformations, showing no apparent preference for any specific configuration.

**Figure 5:**
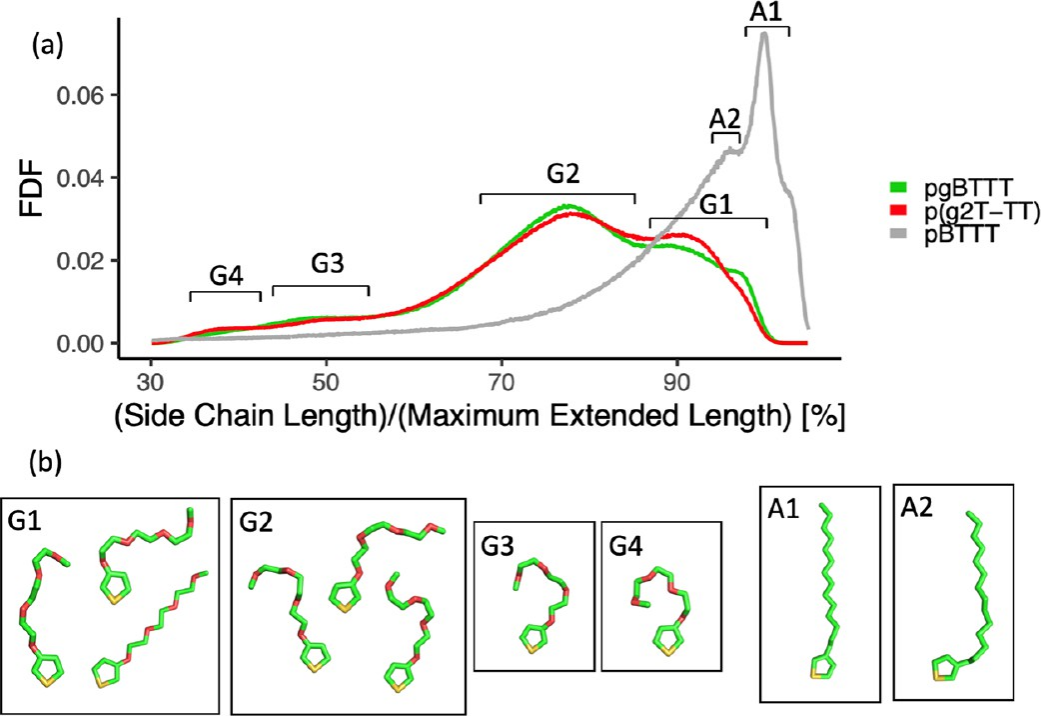
(a) Frequency distribution function (FDF) of side-chain lengths for alkylated and glycolated polymers, normalized by their maximum extended length. The broader range of conformations adopted by the TEG side chains is clearly visible. The side chain conformations were sampled from high coverage MD simulations for each polymer type. (b) Typical conformations associated with the peaks in the FDF.

When simulated by MD, the deposition of pgBTTT and p(g2T-TT) on a Au substrate in the low-density limit resulted in conformations very similar to those seen in STM (Figures S14 and S15). The simulations clearly reproduced the wider range of possible conformations of the TEG side chains, when compared to alkyl side chains of pBTTT. Figure 5 shows the frequency distribution of the side chain lengths, demonstrating that the C_14_ alkyl side chains predominantly adopt one of two straight conformations (A1 or A2), different only in the rotation of a C–C bond at the beginning of the side chain. TEG side chains, on the other hand, show a wide distribution of lengths with broad peaks that can roughly be associated with the number of alternating gauche conformations in the TEG chain. The main conformations G1–G4 closely resemble the shapes of the TEG side chain used to fit the STM images in Figure 4.

Figure 4 further shows that, in the areas of low surface coverage, the backbones of both pgBTTT and p(g2T-TT) polymers are highly kinked. Since the backbones of these polymers are chemically identical to those of pBTTT, the same molecular models of Figure S4 can be used to fit the STM images. Also in this case, this results in an excellent match in terms of backbone conformation and position of the side chains (Figure 4c,d). As with pBTTT, it was possible to gather statistics by fitting multiple STM images. This resulted in a relative frequency of *cis* T–T bonds of 27% and 13% for pgBTTT and p(g2T-TT), respectively, and of *cis* T–TT bonds of 16% and 5% for pgBTTT and p(g2T-TT), respectively (Table S1). The observation that the backbones of p(g2T-TT) appear to be less kinked than those of pgBTTT is likely to arise at least in part from the steric barrier associated with the formation of *cis* T–T bonds in p(g2T-TT) (Figure S5). Also in the MD simulations, the backbones generally adopt a highly kinked conformation, with all simulated single oligomers containing at least one backbone kink, and usually more than two (Figures S14 and S15).

**Figure 6:**
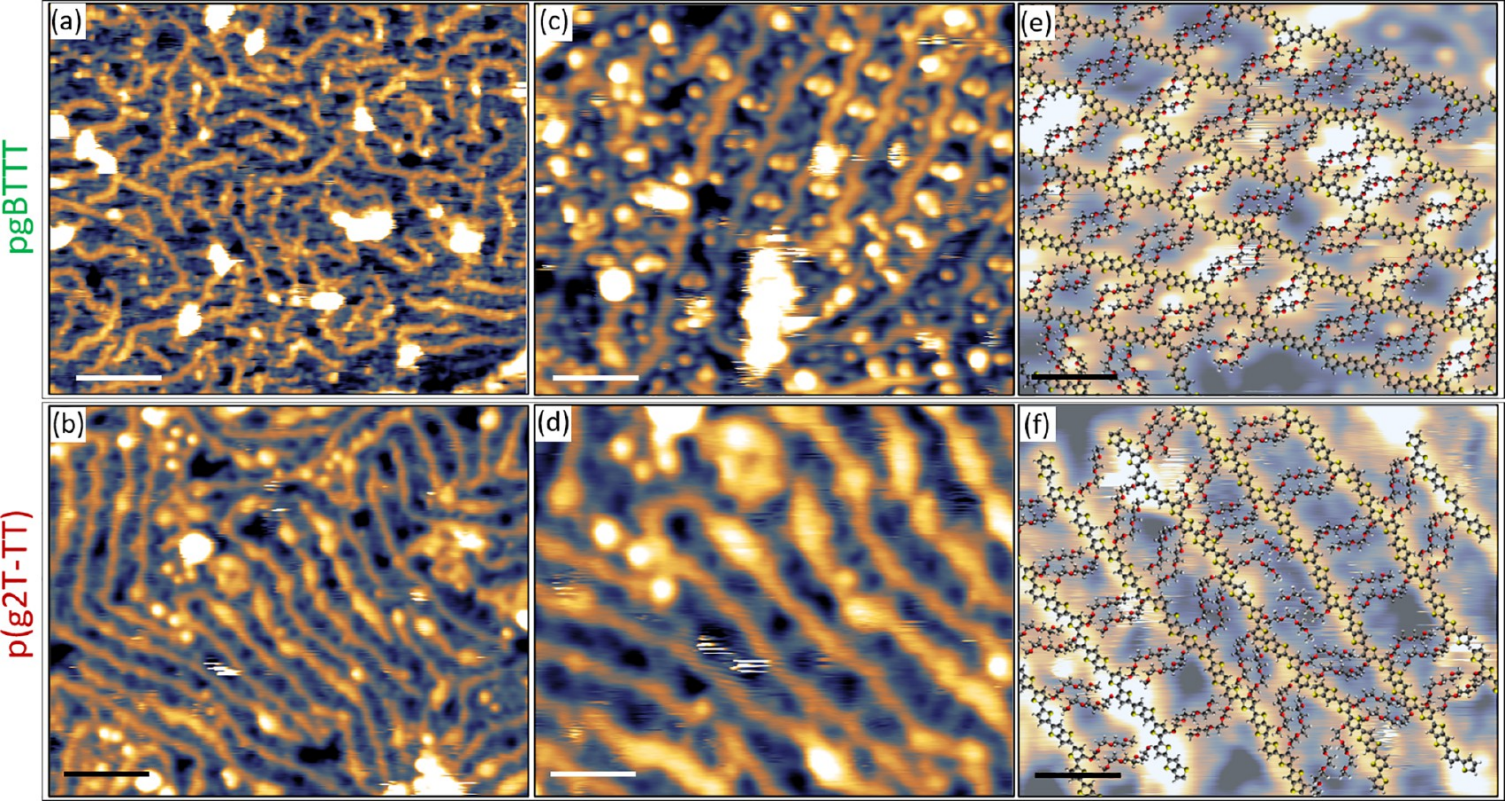
STM images of high surface coverage areas for pgBTTT (upper row) and p(g2T-TT) (lower row) deposited in UHV by ESD on a Au(111) surface. (a) and (b) Large scale images where occasional local parallel arrangements of the backbones can be seen. (c) and (d) Smaller regions where regular, dimmer features, ascribable to TEG side chains, can be observed between neighboring backbones. (e) and (f) High-resolution images with an overlayered molecular model obtained with the procedure described in the text. All images were acquired at 77 K. Scale bars in (a) and (b) correspond to 6 nm; in (c) and (d) to 3 nm, in (e) and (f) to 2 nm. Scanning parameters in constant current mode: (a) −1.5 V, 190 pA; (b) +1.2 V, 80 pa; (c) +1.3 V, 75 pA; (d) +1.2 V, 80 pA; (e) +0.85 V, 90 pA; (f) +1.2 V, 80 pA.

As shown in Figure 6a,b, in areas where the surface coverage is high, both pgBTTT and p(g2T-TT) organize into 2D compact islands. However, the tendency to form an ordered assembly is noticeably lower with respect to pBTTT, with only occasional regions that show the backbones arranging into local parallel structures. Even in these regions, though, the two glycolated polymers display a less regular organization, characterized by a broad distribution of interbackbone distances, with an average value and standard deviation of (15 ± 2) Å for both pgBTTT and p(g2T-TT) (see Figure S7). As will be discussed later, this is most probably the result of the lateral interaction strength of pgBTTT and p(g2T-TT) being weaker than that of pBTTT.

Higher magnification images allow to distinguish regularly spaced features in between neighboring backbones of both glycolated polymers, as shown in Figure 6c,d for pgBTTT and p(g2T-TT), respectively. In particular for pgBTTT, it was possible to achieve a higher resolution which revealed a zigzag pattern within the backbones (similar to that observed for pBTTT) and pairs of double dots on both sides of the backbones. Both features have a periodicity of (13.5 ± 0.5) Å, while the separation of the dots in a pair is (4.5 ± 0.5) Å. These two aspects indicate that the double dots must correspond to paired TEG side chains belonging to neighboring, parallel-aligned polymers. The superposition of pgBTTT molecular models further shows that, irrespective of their actual configuration, the side chains within one of these pairs are at a distance from each other such that they must be directly interacting.

It is evident that the surface self-assembly of both alkylated and glycolated polymers is driven by the interaction of their side chains. However, because of the different chemical structure of alkyl and EG chains, these interactions are of a different nature and strength. For alkylated polymers, the driving force is the maximization of the vdW contacts between the aliphatic side chains, which is achieved when they are in a straight, all-*trans* conformation and at a separation approximately corresponding to the structure and density of crystalline PE. As discussed above, this is described well by the model proposed by Kline et al.,^17^ which assumes that the alkyl side chains aim to reach the assembly of unconstrained PE chains. What we propose here is that EG side chains behave in an analogous manner, that is, that they also try to attain a conformation resembling the assembly of their free analogues, i.e., polyethylene glycol (PEG). PEG is normally a liquid or an amorphous solid, depending on molecular weight, but samples with a highly homogeneous chain length can actually crystallize. In particular, it was reported that uniform samples of PEG_16_ organize into crystalline domains of nested antiparallel helices with a separation of 4.5 Å.^43^ As mentioned above, also TEG chains can assume a helical conformation when all oxygen atoms are gauche to each other and are rotated in the same direction (Figure S6b). The 11-atom TEG chains are not long enough to develop the extended intermolecular interactions that characterize the crystalline form of PEG_16_, but we propose that they create short bonding patterns that locally resemble them.

In order to test this hypothesis, we considered TEG chains in a helical configuration—specifically, pairs of PEG_16_ chains in the X-ray diffraction structure^43^—as a starting point for devising possible conformations of interacting TEG side chains of pgBTTT and p(g2T-TT) (see section 9 in the SI). Two configurations in particular were found to maximize side chain-to-side chain overlap (Figure S8e,f), while all others had a lower degree of superposition (one example given in Figure S8g). A selection of these pairs of TEG side chains was then used to build models of full pgBTTT and p(g2T-TT) polymers aligned parallel to each other and interacting through their side chains (Figures S9 and S10 for pgBTTT and p(g2T-TT), respectively). Depending on which pair of TEG side chains was chosen and on their orientation, different interstrand distances were obtained in the range between 1.2 and 2.0 nm (Figures S9 and S10). These models were then fitted onto high-resolution STM images of pgBTTT and p(g2T-TT) by following the procedure described in section 10 of the SI (Figures S11 and S12). The resulting fits are shown in Figure 6e,f for pgBTTT and p(g2T-TT), respectively, demonstrating a very good agreement with the STM data and allowing to satisfactorily identify all features in the images.

**Figure 7:**
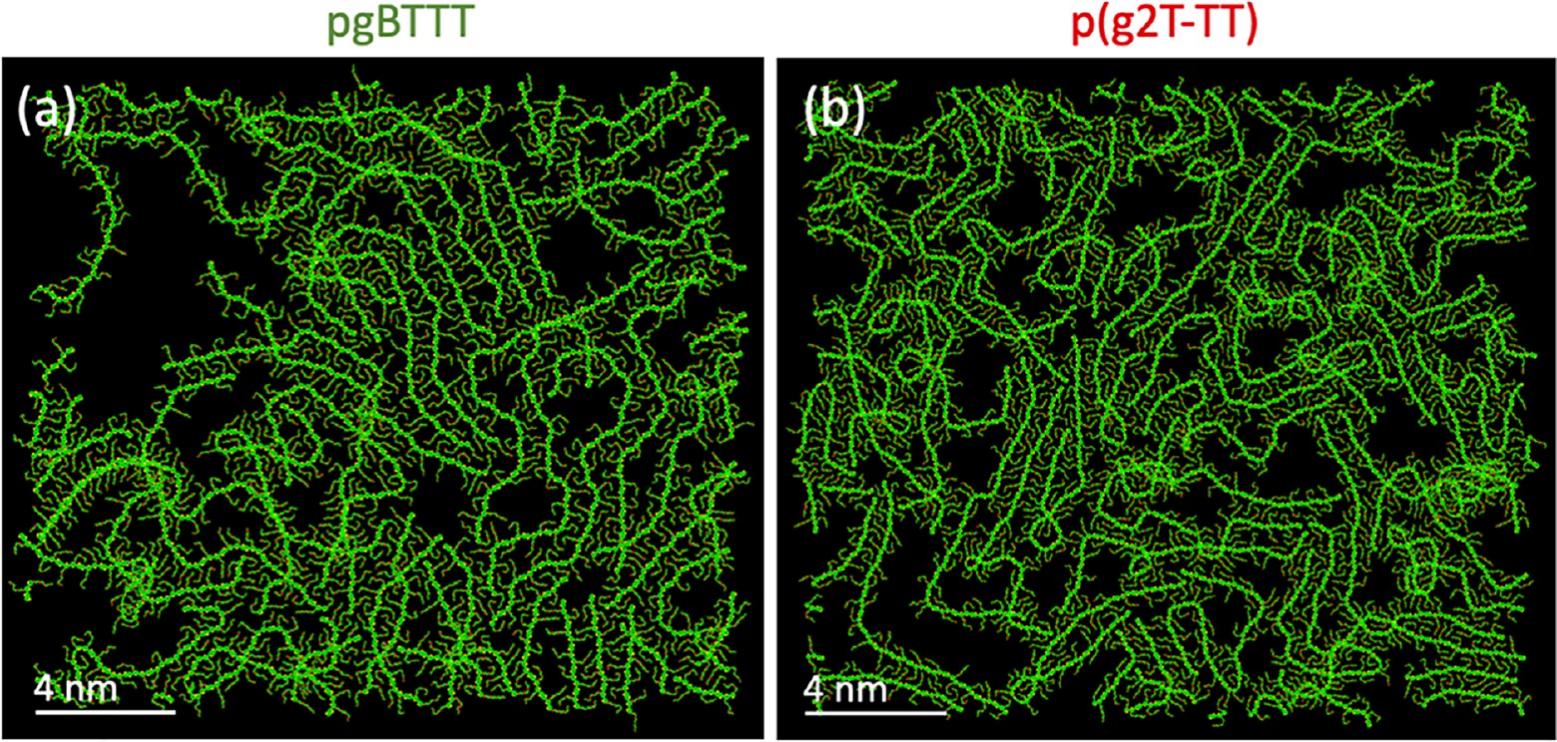
Representative snapshot of MD simulations of high-density pgBTTT (a) and p(g2T-TT) (b) deposited on an Au substrate and evolved for 5 ns. The images clearly show the relative disorder of the assemblies formed by the glycolated polymers when compared to pBTTT.

MD simulations of high-density pgBTTT and p(g2T-TT) deposited on a Au surface show the same qualitative behavior as seen in the STM images (Figure 7). Overall, a greater level of disorder is observed when comparing with the analogous simulations of pBTTT, with a lower proportion of the backbones lying parallel to each other. The glycolated polymer backbones also cross more frequently than in the case of pBTTT, a difference that can be qualitatively seen in the STM images too. Furthermore, also in the simulations, TEG side chains of neighboring polymers are often found to form pairs (Figure S16), with one side chain curling around the other. While these pairs resemble those assumed for interacting free TEG chains as discussed in section 8 in the SI, it should be noted that their conformation is essentially flat and parallel to the surface, while the TEG chains in Figure S8e–g are strongly 3D (having been extracted from the nested helices of PEG_16_^43^). The exact structure that the glycol side chains assume when adsorbed on the Au surface will be influenced by their interaction with the substrate, which is accounted for in the MD simulations. Thus, the configurations of the paired side chains in Figure S16 might represent a flattened version of the structures that free TEG side chains take when interacting with one another in 3D thin films of pgBTTT and p(g2T-TT) and are expected to be a better description of the structures observed in the STM measurements.

The main aspect emerging from the experimental and theoretical analysis of the assembly of pgBTTT and p(g2T-TT), which is in stark contrast with what happens for pBTTT, is that the side chains of neighboring polymers organize in pairs instead of forming an equispaced and fully interdigitated sequence. Since we are assuming that the same fundamental principle governs the lateral assembly of alkylated and glycolated polymers, i.e., their side chains trying to reach their “free-assembly” configuration, the reason for this difference must be sought in the different nature of interactions between the side chains. The pBTTT aliphatic side chain interactions are dominated by vdW forces, which are attractive and smoothly varying along the length of the chain. This results in a straight, all *trans* geometry being the lowest energy configuration for interacting alkyl chains. The ideal assembly is achieved by tilting the side chains with respect to the backbones and thus optimizing their mutual distances^17^ and by interpenetrating each other as far as possible, thus maximizing their overlap. Both requirements can be easily achieved in the case of PE chains, thanks to the lenient characteristics of the dominant vdW interactions among them.

On the contrary, due to the presence of the electronegative and thus partially negatively charged oxygen atoms, the interaction between EG chains is a combination of electrostatic (both attractive and repulsive) and vdW forces and strongly varies along the chain. As a consequence, a helix instead of a straight chain is the optimal configuration for free but interacting EG side chains. However, attaining the optimal assembly between these helices where each oxygen atom is surrounded by methylene groups^43^ is not so simple for EG chains constrained by being anchored to the backbones of the polymers at a fixed distance from each other. This might be expected to result in a weaker effective lateral interaction strength between pgBTTT and p(g2T-TT) polymers with respect to the case of pBTTT. Experimental observations of liquids of PEG and PE can provide some insight into the relative interactions that play a role when they are used as side chains. In particular, PE is observed to have a higher melting point than PEG.^44,45^ Moreover, viscosity is also often used as an indirect measure of intermolecular interactions.^46^ While measurements of PE and PEG viscosity are difficult to obtain at the same temperature (due to the different melting points), literature does seem to suggest that the viscosity of PE is greater than that of PEG.^47,48^ Both melting point and viscosity measurements are thus consistent with our observation that PE interpenetrates with other PE side chains more readily than PEG does with itself.

The final observation that emerges from comparing the high surface coverage areas of pBTTT with those of its glycolated analogues concerns the conformation of the polymer backbones. While a detailed discussion of the frequency of *cis* and *trans* bonds involves also steric considerations (see Figure S5), an overall analysis of the experimental data shows that pBTTT is characterized by a frequency of backbone kinks that is lower than what would be expected from looking at the torsional potential energies. In fact, due to the formation of intramolecular S···O bonds, the DFT calculated torsion energies of pBTTT, pgBTTT, and p(g2T-TT) predict a higher rotational rigidity for glycolated polymers than for their alkylated equivalent (Figure S1). Thus, pgBTTT and p(g2T-TT) should have straighter backbones than pBTTT. This does not agree with the results of the measurements, where the frequency of backbone kinks in pBTTT is seen to be lower than that of pgBTTT and comparable to that of p(g2T-TT) (Table S1). In order to rationalize this apparent discrepancy, it is important to recall that, for laterally interacting polymers, the energetics of a given backbone conformation is not only determined by its torsional potential energy but also by the degree of side chain coupling this conformation allows. In particular, backbone kinks cause losses of side chain interdigitation and thus reduce intermolecular interaction energy. Since the lateral interaction strength of pgBTTT and p(g2T-TT) is lower than that of pBTTT, the loss of side chain interdigitation bears a stronger energetic cost for pBTTT than for its glycolated analogues. In other words, since intermolecular interactions are stronger for polymers with alkyl side chains, they drive the backbone conformations more significantly than in polymers with glycolated side chains. A similar argument also explains why the degree of kinking of pgBTTT and p(g2T-TT) does not depend much on whether the polymers are isolated or mutually interacting (see Table S1). We expect the same phenomenon to be true also for the assembly of 3D polymer films, representing a further confirmation that the interaction between side chains plays an essential role in determining the microstructure of conjugated polymers, not only for the way that polymer lamellae interact among each other but also in terms of conformation of the backbones.

## Conclusion

In this combined experimental and theoretical study of surface-adsorbed conjugated polymers, we investigated the effect of replacing alkyl side chains with glycol ones on the polymer assembly and microstructure. The measurements were performed by STM on polymers vacuum-deposited onto gold and silver surfaces by ESD and allowed us to determine molecular-scale details of the assemblies’ microstructure. The MD simulations show an excellent agreement with the experimental data, thereby extending our insight into the polymers’ conformation and assembly to the atomic scale. Moreover, the fact that these structures spontaneously developed from a range of disordered conformations that had been pre-equilibrated for a short time in vacuum also provides a further validation of the used force field.

The main result of our work is that for both alkylated and glycolated polymers, both the assembly and the conformation are profoundly determined by the interaction of their side chains. In order to rationalize these observations, we propose that in both cases the driving force for assembly is the attempt of the side chains to attain the conformation of their free analogues, i.e., the assembly of polyethylene and polyethylene glycol, respectively. We show that the main difference between the microstructure of the two polymers and the conformation of the individual polymer chains can indeed be traced back to the different interaction patterns and energies of PE and PEG.

While our study identifies specific features of the observed assembly that are influenced by the molecule–substrate interaction, several others are independent of the presence of a surface and are thus expected to directly translate to the 3D packing of these polymers into “dry” thin films, i.e., solid films that are formed by drop casting or spin coating after evaporation of the solvent. Direct examples are the structural parameters determined by STM in 2D that are found to closely match those measured by GIWAXS in 3D, for both alkylated and glycolated polymers (e.g., the lamellar separation and the distance between side chains). By extending this similarity, we can use the results of this work as a source of detailed, subnm-scale information on the microstructure of conjugated polymers, which has been inaccessible so far. In particular, in the following, we list the main features that we have observed in our measurements and simulations of 2D monolayers and that we expect to hold in a qualitatively similar manner in 3D thin films. (i) Conjugated polymers with alkyl side chains assemble in more extended and more regular and crystalline domains than their counterparts with glycol side chains. This is because the interaction energy between PE chains (only attractive vdW forces, smoothly varying along the chains) is stronger than that between PEG chains (combination of attractive and repulsive vdW and electrostatic forces, strongly varying along the chains). (ii) Alkyl side chains adopt an extended all-*trans* conformation, and their orientation with respect to the backbones is highly regular. On the contrary, the EG side chains display a high variability with an overall much more twisted conformation, characterized by several gauche oxygen atoms. (iii) When interacting with each other, alkyl side chains tend to maximize their overlap, resulting in highly interdigitated polymer assemblies. Mutually interacting EG side chains can also interdigitate, but due to the different type of forces at play and the smaller energetic penalty associated with the loss of interdigitation, they display a high variability in the type and extent of overlap, which is reflected in larger variation of the polymer interbackbone distances (lamellar spacing in 3D). (iv) Despite having the same attachment density along the backbones, the aliphatic side chains of interacting pBTTT polymers form homogeneously interdigitated and equispaced sequences, while the EG side chains of pgBTTT and p(g2T-TT) organize into pairs. In fact, for the alkylated polymers, the optimal distance between the side chains (∼4.7 Å in PE^17^) is attained by tilting them with respect to the backbone. While this can be done without any major impediment in pBTTT, this is not the case for the EG side chains of the glycolated polymers, since their twisted conformation and nonuniform interaction potential cause significant steric limitations and a reduced overlap. Such limitations allow only a few possible tilt angles to be compatible with the ideal side chain separation (∼4.5 Å in PEG^43^) and result in a paired side chain assembly, even if this causes an overall loss in interaction energy. We propose that taking this observation into account when designing new glycolated side chain polymers might result in more ordered microstructures. For example, choosing the spacing of side chain attachment points on the polymer backbone, so as to allow side chains from neighboring backbones to interdigitate with a separation close to their optimum of ∼4.5 Å, should strengthen the polymer lateral interactions and thus produce a more ordered microstructure. (v) The conformation of the polymer backbones is not only controlled by their torsional potentials but also by the optimization of side chain interactions. The relative weight of these two contributions is determined by the side chain coupling strength which can significantly overcome the effect of the torsional potential, as happens for polymers with linear alkyl side chains.

Finally, we notice that the choice to focus our study on prototypical model-system polythiophenes ensures that several of our results are of fundamental nature and go beyond the specific systems we examined here. We thus expect our main conclusions to have a potential impact on a wide class of conjugated polymers, especially in terms of the similarities and differences between alkylated and glycolated polymers. This is particularly important for the latter class of materials, which is gaining increasing prominence due to its promising performance in bioelectronic and thermoelectric applications, but whose microstructure is currently still not well characterized and understood and for which detailed structure–function relationships are still lacking.

## Methods

### Scanning Tunneling Microscopy

Samples have been prepared by electrospray deposition (4-stage Molecularsprat Ltd. system) of a solution of the polymers dissolved in chlorobenzene (≈ 0.025 g/L) and diluted with methanol in a 4:1 volume ratio. The substrates were kept at room temperature during ESD with the deposition ion current monitored. The total deposition charges amounted to 8 pAh for pBTTT and 6 pAh for pgBTTT and p(g2T-TT). Films of Au(111)/mica and Ag(111)/mica (Georg Albert PVD, 300 nm thickness) were used as substrates and prepared in UHV by cycles of argon sputtering and annealing to 500 °C. STM measurements of all polymers were performed in UHV. pBTTT was measured with a variable-temperature STM (SPECS Aarhus), with the sample cooled to −145 °C, while the glycolated polymers were measured on a low-temperature STM (CreaTec Fischer & Co. GmbH), kept at −196 °C. All images were acquired in constant current feedback mode.

### Molecular Dynamics Simulations

MD simulations were performed using a force field based on OPLS-AA,^49^ with interthiophene dihedrals parametrized to fit with DFT at the B3LYP/6311G** level of theory.^50^ Glycol side chain parameters are from work by Woods et al.^51^ and are based on OPLS-aa atom types, with other backbone bonded parameters from work by Moreno et al. and Bhatta et al.^52,53^ The force field used is closely related to that presented in a recent MD simulation study of oxy-bithiophene oligomers by Siemons et al.^54^

All simulations were performed with 20-mers on a Au substrate, with Au parameters from work by Su et al.^55^ As a general procedure, all simulations begin with an in-vacuum energy minimization. Either single or multiple polymers are then placed 3 Å from the Au surface. After beginning the NVT simulation, they are quickly adsorbed onto the surface, after which they equilibrate into a final conformation. For simulations of high density areas of the polymer, a subsequent simulation in NPT is performed. All simulations were performed in Gromacs 2018.2.^56,57^

## Data Availability

The datasets generated during and/or analyzed during the current study are available from the corresponding author on reasonable request.

## Supporting Information Available

Further details on the experimental and theoretical methods, STM images displaying the molecular order in high and low coverage regions of pBTTT, description of the molecular models used for fitting the STM images, analysis of the kinks in the polymer backbones, detailed experimental statistics of cis and trans bonds in the polymer backbones, possible TEG chain configurations, experimental statistical analysis of the interbackbone distance distributions in high-density areas for pgBTTT and p(g2T-TT), description of the models used to fit the STM images of interacting glycolated side chains, description of the fitting procedure of molecular models on STM images for pgBTTT and p(g2T-TT), details of the MD simulations of pBTTT, pgBTTT and p(g2T-TT) (PDF)
