# Supplementary material for: The Effect of Glycol Side Chains on the Assembly and Microstructure of Conjugated Polymers
Source: ACS Nano. 2022 Dec 14;16(12):21303–14. doi: 10.1021/acsnano.2c09464 (PMC9798861; doi:10.1021/acsnano.2c09464)
Supplement: Supplementary file 1 — nn2c09464_si_001.pdf [file nn2c09464_si_001.pdf]

Supporting Information

The effect of glycol side chains on the assembly and microstructure of conjugated polymers

Stefania Moro,[1,2] Nicholas Siemons,[3] Oscar Drury,[1] Daniel A. Warr,[1] Thomas A. Moriarty,[1] Luís M.A. Perdigão,[1] Drew Pearce,[3] Maximilian Moser,[4] Rawad K. Hallani,[5] Joseph Parker,[1] Iain McCulloch,[4,5] Jarvist M. Frost,[3] Jenny Nelson[3] and Giovanni Costantini[1,2] *

[1]*Department of Chemistry, University of Warwick, Coventry, CV4 7AL, United Kingdom.*

[2]*School of Chemistry, University of Birmingham, Birmingham, B15 2TT, United Kingdom.*

[3]*Department of Physics, Imperial College, London, South Kensington, London, SW7 2AZ, United Kingdom.*

[4]*Department of Chemistry, University of Oxford, Oxford, OX1 3TA, United Kingdom.*

[5]*Physical Science and Engineering Division, King Abdullah University of Science and Technology (KAUST), Thuwal, 23955-6900, Saudi Arabia.*

* email: g.costantini@bham.ac.uk

Table of Contents

1. Methods....2
1.1. Scanning tunnelling microscopy....2
1.2. Molecular dynamics simulations....3
2. Order in high coverage regions of pBTTT....5
3. Low surface coverage regions of pBTTT....5
4. Molecular models used for fitting the STM images....6
5. Kinks in the polymer backbones....7
6. Statistics of *cis* and *trans* bonds in the polymer backbones....8
7. TEG chain configurations....10
8. Inter-backbone distance distributions in high-density areas for pgBTTT and p(g2T-TT)....10
9. Models of interacting glycolated side chains....11
10. Fitting of molecular models on STM images for pgBTTT and p(g2T-TT)....13
11. Details of the MD simulations....15
References....17

1. Methods

1.1. Scanning tunnelling microscopy

Measurements were performed on two ultrahigh vacuum (UHV) systems: a variable temperature (VT) and a low temperature (LT) scanning tunnelling microscope (STM), each equipped with an electrospray deposition (ESD) setup. The samples were dissolved in chlorobenzene at concentrations ≈ 0.025 g/L, and methanol was added to a 4:1 volume ratio. The molecules were deposited from solution by ESD with a 4-stage Molecular Spray system. The deposition current was monitored on the target substrate and typical deposition charges were 8 pAh for pBTTT and 6pAh for pgBTTT and p(g2T-TT). Au(111)/mica and Ag(111)/mica thin films (Georg Albert PVD, 300 nm thickness) were used as substrates and prepared in UHV by cycles of Argon sputtering (1 kV) and annealing to 500 °C. The measurements for pBTTT were performed on Au(111) and Ag(111) substrates, while all measurements for pgBTTT and p(g2T-TT) were carried out on Au(111). The substate did not affect the overall assembly of the material that formed similar structures on both surfaces. The VT-STM (SPECS Aarhus) was

cooled with a flow liquid nitrogen to a temperature of –145 °C, while the LT-STM (CreaTec Fischer & Co. GmbH) was kept at –196 °C by a liquid nitrogen bath cryostat. The first instrument was used to acquire the images for pBTTT, the second one to obtain the data for pgBTTT and p(g2T-TT). All images were acquired in constant current feedback mode with an etched tungsten tip that was treated by argon sputtering after insertion in UHV, to remove the oxide. The STM images were processed with WSxM[1] and Gwyddion,[2] the molecular models were made and optimised in Avogadro,[3] and their comparison with/superposition on the STM images was performed with the LMAPper software.[4]

1.2. Molecular dynamics simulations

The used molecular dynamics (MD) force field is based on OPLS-AA,[5] with inter-thiophene dihedrals parameterised to fit with density functional theory (DFT) at the B3LYP/6311G** level of theory,[6] and can be seen in Fig. S1. . It should be noted that in the DFT simulation the side chains were approximated by a simple methyl group for pBTTT and by a hydroxy group for pgBTTT and p(g2T-TT). This explains why the potential energy surfaces for the T-T dihedrals are essentially identical for pBTTT and pgBTTT in Fig. S1.

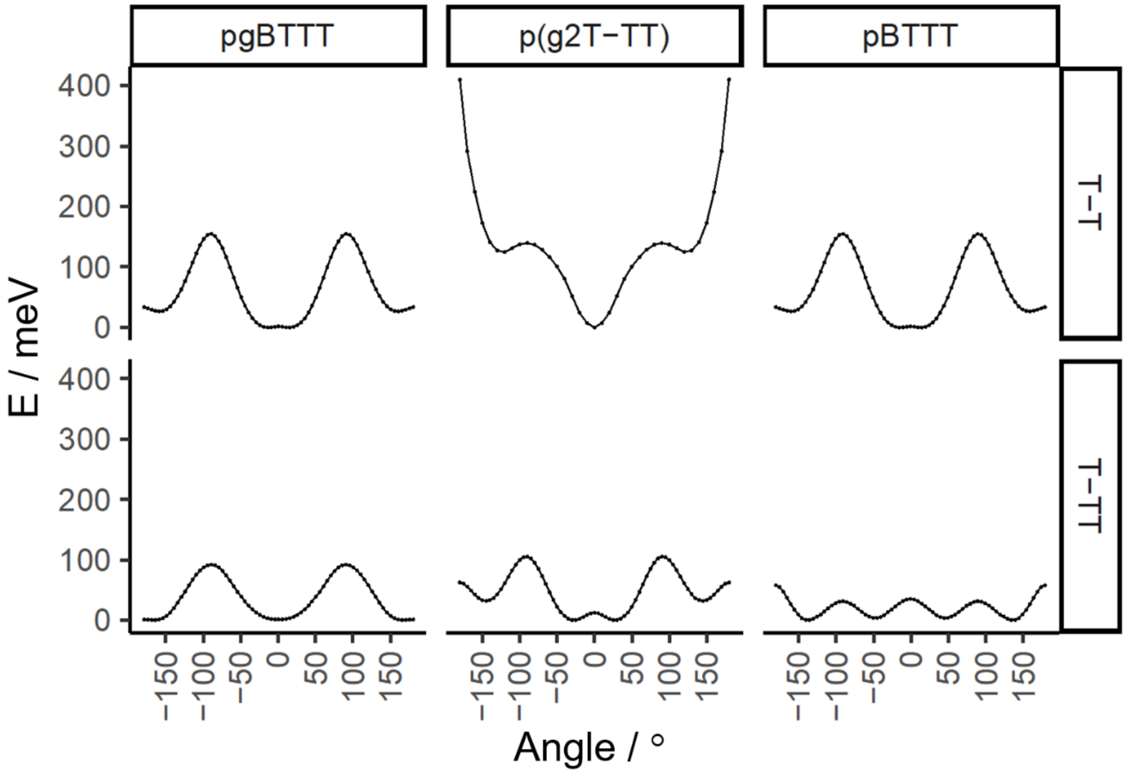

Figure S1 Dihedral potential energy surfaces used in the MD simulations, for both the thiophene-thiophene (T-T), and thiophene-thienothiophene (T-TT) dihedrals.

Glycol side chain parameters are from work by Woods *et al.*[7] and are based on OPLS-aa atom types, with other backbone bonded parameters from work by M. Moreno *et al.* and R. Bhatta *et al*.[8,9] The force field used is closely related to that presented in a recent MD simulation study of oxy-bithiophene oligomers by N. Siemons *et al.,*[10] and it is therefore expected that the validation performed on that force field is also valid for the one used in this study. For the full force field parameterisation, please see the public GitHub Repository.

Simulations were performed using GROMACS2018.2.[11,12] Electrostatics and Van-der-Waals forces are computed using a cut-off scheme. Particle-Mesh Ewald Summation, whilst more accurate for bulk systems, scales poorly with vacuum space and hence is avoided here. Hydrogen bonds are constrained using a LINCS algorithm, allowing for 2 fs time steps. Initial configurations were prepared using GROMACS 2018.2 and Packmol.[13] Trajectories were analysed using PyMOL.[14] Initial energy minimisation was performed on all simulations using the steep integration method and a time step of 1 fs, until the change in energy was less than numerical accuracy. The gold substrate was modelled using a 12-6 Van-der-Waals potential with parameters obtained from.[15]

All simulations were performed with 20-mers on a Au substrate, with Au parameters from work by L. Su.[16] In all simulations, initial configurations were energy-minimised in vacuum and placed 3 Å above the surface of the substrate, measured from the substrate to the centre of mass of the oligomer. During the subsequent NVT simulations, the oligomers were deposited onto the substrate, being driven by their Van-der-Waals interaction, after allowing for some in-vacuum equilibration. For simulations of low molecular density areas, oligomers were simulated in NVT for 5 ns using a velocity-rescale algorithm for temperature coupling.[17] For simulations of high molecular density areas, after vacuum energy minimisation, 48 oligomers were placed 3 Å above a 60 nm × 60 nm surface. The deposition occurred during the 5 ns NVT simulations, which were followed by a subsequent 20 ns NPT simulation that was performed using semi-isotropic pressure coupling to apply pressure only parallel to the substrate. For NPT, pressure coupling is ensured using a Parrinello-Rahman barostat.[18,19]

To ensure sufficient statistical sample, all simulations of single oligomers were performed over 36 replicas. Simulations of multiple polymers were performed over 4 replicas, each with 48 polymers.

2. Order in high coverage regions of pBTTT

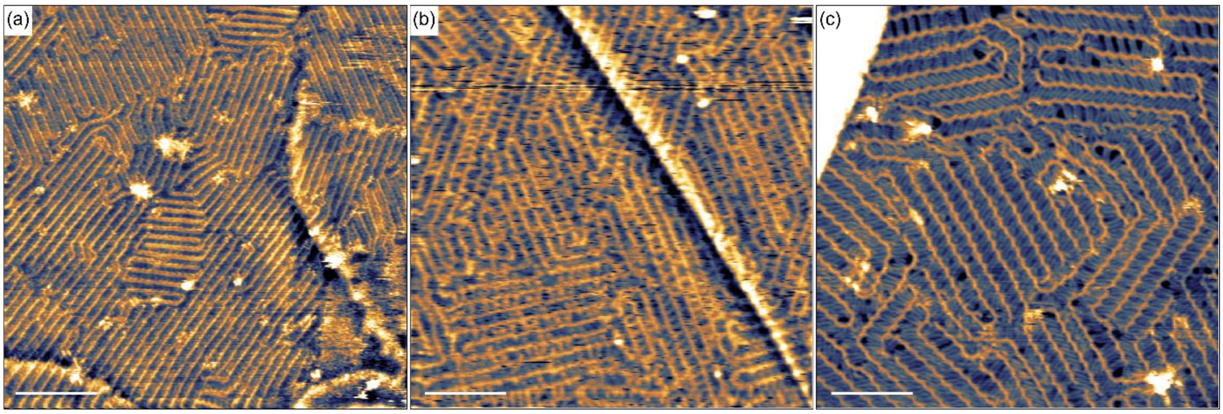

Figure S2. STM images of pBTTT on Au(111) showing the highly regular assembly of pBTTT at high coverages, with wide areas of parallel backbones observed. Scale bars correspond to a) 4 nm, b) 3 nm and c) 2 nm.

3. Low surface coverage regions of pBTTT

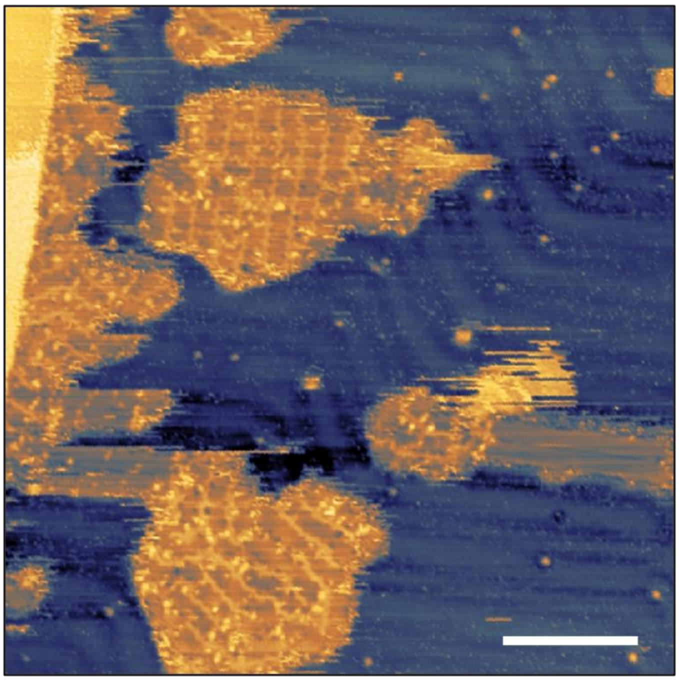

Figure S3. STM image of pBTTT on Au(111) showing that, even in areas of lower local polymer coverage, pBTTT forms compact two-dimensional (2D) islands, leaving areas of bare exposed substrate. The wavy appearance of the gold surface is given by the Au(111) herringbone reconstruction.

4. Molecular models used for fitting the STM images

The molecular models used to fit the polymers in the STM images by means of the LMAPper software were created in the Avogadro molecular editor. Separated models were used for the side chains and for the backbone and their configuration was locally minimised with the MMFF94 force field of Avogadro to find optimal bond lengths and dihedral angles. In particular, the models of the alkyl side chains were based on isolated C_{14} chains in their fully extended linear configuration while different configurations of the triethylene glycol (TEG) sidechains were used with various degrees of chain bending (see Section 7 of the SI).

For the backbones, fragments based on isolated monomer and dimer repeat units of pBTTT where considered, where the side chains were substituted by methyl groups. The corresponding Avogadro minimised structures had a planar configuration of the conjugated backbones with thiophene-thiophene (T-T) and thienothiophene-thiophene (T-TT) linkages in a *trans* relative orientation with respect to the orientation of the sulphur atoms (see Fig. S4ai). In order to account for different configurations of the polymer backbones, and in particular for the possibility of kinks, isolated pBTTT monomer and dimer repeat units with the T-T and T-TT linkages in a *cis* relative orientation (i.e. where a 180° rotation was inserted around T-T and T-TT single C-C bonds, with respect to the all-*trans* lowest energy configuration) were also considered. All these individual models where then composed together in LMAPper in order to best fit the features appearing in the STM images.

The structure of a few of these models was also optimised in MD to check for a qualitative agreement between the force field used for the MD, and that used for the geometry optimisations. For the case of planarised units (such as those adsorbed on the gold surface), the geometry optimised structures for both the MD force field and the MMFF94 force field showed close qualitative agreement. It is important to note however, that the planarised structures modelled in this study, do not correspond to minimum-energy ‘free structures’ (i.e. not adsorbed onto the gold surface), as many dihedrals do not have their minima at 0°.

5. Kinks in the polymer backbones

Based on the molecular models created in Avogadro, it was found that any significant kink in the polymer backbone must arise because of one or more T-T bonds in a *cis* configuration (see Fig. S4). This is due to the fact that the T-T bonds are oriented at an angle of about 25° with respect to the overall straight direction of the backbone (see Fig. S4b). On the other hand, the T-TT bonds are almost parallel to the backbone axis, so that their *cis* configuration does not produce as much of a distinct kink in the backbone (Fig. S4biii).

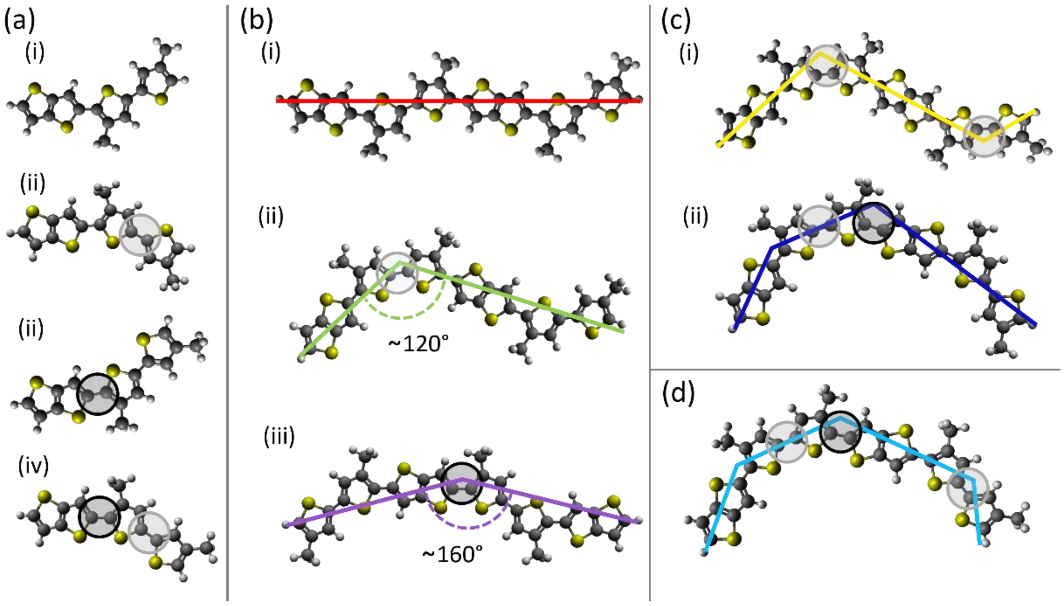

Figure S4. Possible configurations of monomers and dimers of pBTTT obtained by 180° rotations around T-T and T-TT bonds. For simplicity, the actual side chains of pBTTT have been substituted for methyl groups. The backbone configurations are the same also for pgBTTT and p(g2T-TT), but the side chains attachment positions shown here correspond to the 2-2' positions of pBTTT and pgBTTT. (a) All possible configurations of a single BTTT monomer unit. Black circles indicate a *cis* configuration of a T-TT bond, grey circles a *cis* configuration of a T-T bond. (b) All possible configurations of a BTTT dimer for the case of (i) all-*trans* bonds, (ii) one *cis* T-T, and (iii) one *cis* T-TT bond. The straight sections of the backbone are illustrated by straight lines and their intersections demonstrate the formation of kinks. Different colours for the straight segments correspond to different kinks. (c) Selected configurations for two *cis* bonds in a pBTTT dimer. (d) Example of the sequence of three *cis* bonds that create an almost 180° bend in the backbone. Further configurations are possible but only produce changes in the side chains positioning along the backbones with smaller modifications the overall backbone geometry. The models were created and energy-optimised by using the MMFF94 Avogadro force field.

6. Statistics of *cis* and *trans* bonds in the polymer backbones

A statistic of the backbone conformation was collected from the experimental STM data following two different procedures, depending on the resolution of the image. When possible, the polymers were fully fitted with optimised molecular models for both the backbones and the side chains (see Section 4). This allowed to estimate the number of both T-T and T-TT *trans* and *cis* conformations. An example of this procedure is shown by the fitted images in Figs. 1c and 1d, Fig. 4 and Figs. 5e and 5f. On the other hand, when the resolution of the STM images was not high enough to allow a detailed and complete fit but sufficient that backbone kinks could be recognised, these latter were simply counted in the images, as they are equivalent to the number of *cis* T-T bonds. As can be seen from Fig. S4biii, the angle introduced by a *cis* T-TT bond is smaller and therefore harder to detect with certainty, so the count for the *cis* T-TT bonds was not included in this case. The combined results of the two methods are reported in Table S1. The total number of T-T and T-TT bonds was evaluated by direct counting in the first procedure, adopted for high-resolution images. For lower resolution images, the total length of each evaluated polymer was measured and divided by the length of the pBTTT repeat unit; this ratio is a good estimation of the total number of T-T and T-TT bonds. Note that no data are present in Table S1 for isolated pBTTT, as no such polymer has been observed.

Table S1. Frequencies of *cis* and *trans* bonds in the backbones of surface adsorbed pBTTT-C_{14}, pgBTTT and p(g2T-TT) polymers, based on the analysis of STM images. The total frequency of kinks is the sum of the frequencies of *cis* T-T and *cis* T-TT bonds.

		total T-T	*cis* T-T	*cis* T-T (%)	total T-TT	*cis* T-TT	*cis* T-TT (%)
pBTTT-C_{14}	isolated	–	–	**–**	–	–	**–**
	assembled	592	62	**10.4**	1114	82	**7.4**
pgBTTT	isolated	495	133	26.9	633	96	15.6
	assembled	214	47	**22.0**	343	46	**13.4**
p(g2T-TT)	isolated	448	57	12.7	670	33	4.9
	assembled	385	29	**7.5**	688	32	**4.7**

The differences in kink frequencies between pBTTT and its glycolated derivatives and the role played by intermolecular interactions in determining this, are discussed in the main paper. Here we only note that a *cis* T-T bond in p(g2T-TT) is accompanied by a significant steric clash between the TEG side chains of the corresponding thiophenes, while this is not the case for pgBTTT (see Fig. S5). This is because, being attached to the 3 and 3' positions, the TEG chains are closer together in p(g2T-TT) than in pgBTTT. As a consequence, when restricting the comparison to p(g2T-TT) and pgBTTT, one would expect a lower frequency of *cis* T-T bonds in the former polymer than in the latter, as is effectively observed in the experiments (see Table S1). *Cis* T-TT bonds do not cause any steric clash (see Fig. S5), but DFT calculation predicted a slightly higher torsional potential energy for p(g2T-TT) than for pbTTT (see Fig. S1).

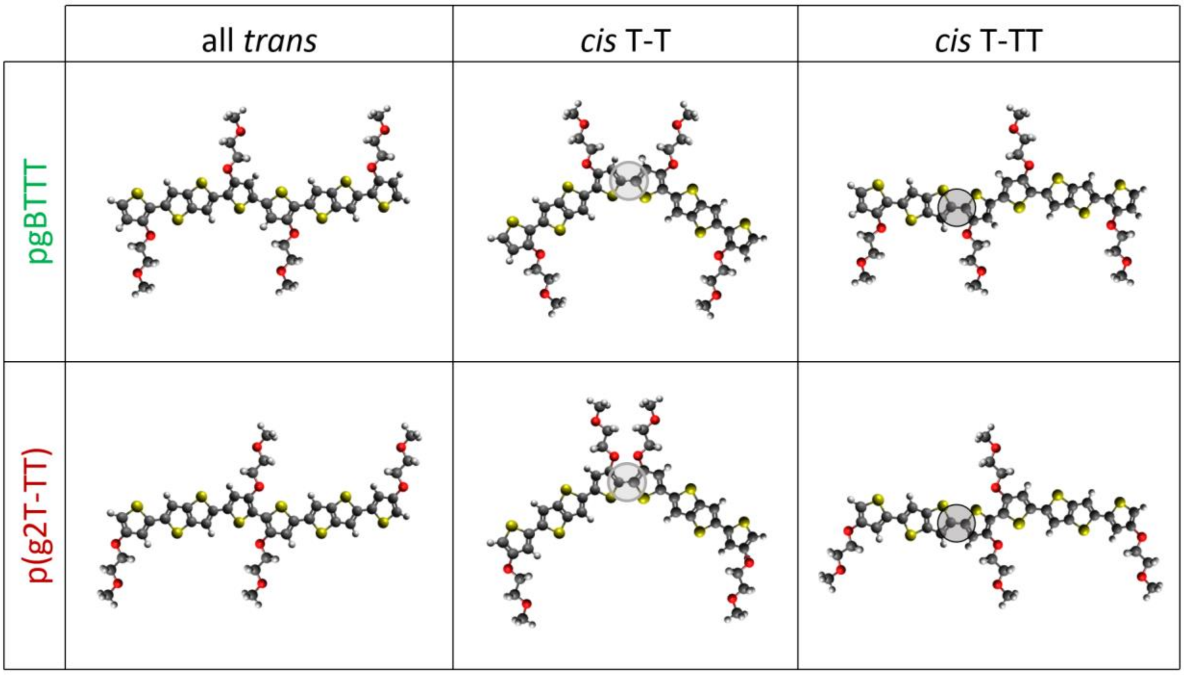

Figure S5. Conformations of pgBTTT and p(g2T-TT) dimers for all *trans* bonds, one *cis* T-T and one *cis* T-TT bond. Grey and black circles highlight a *cis* T-T and T-TT bonds, respectively. For simplicity, the polymer side chains are represented by EG instead of full TEG groups.

7. TEG chain configurations

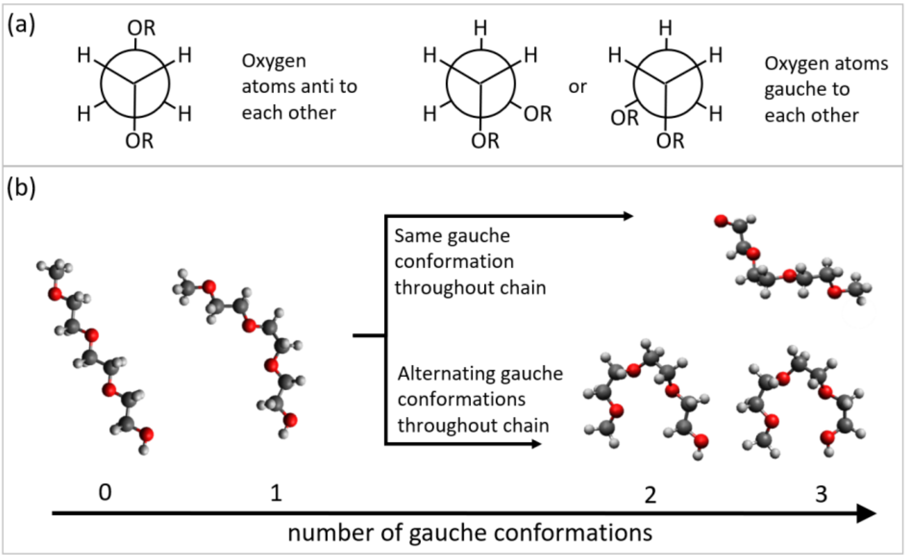

Figure S6. Possible configurations of TEG chains as a function of the number and type of anti or gauche conformation.

8. Inter-backbone distance distributions in high-density areas for pgBTTT and p(g2T-TT)

Inter-backbone distances have been evaluated in high-coverage areas for a series of STM images where locally collinear backbones were observed. Regions of the STM images where three or more polymer backbones were seen to be parallel to one another were chosen to collect a statistics of the distances between backbones, measured normally to the direction

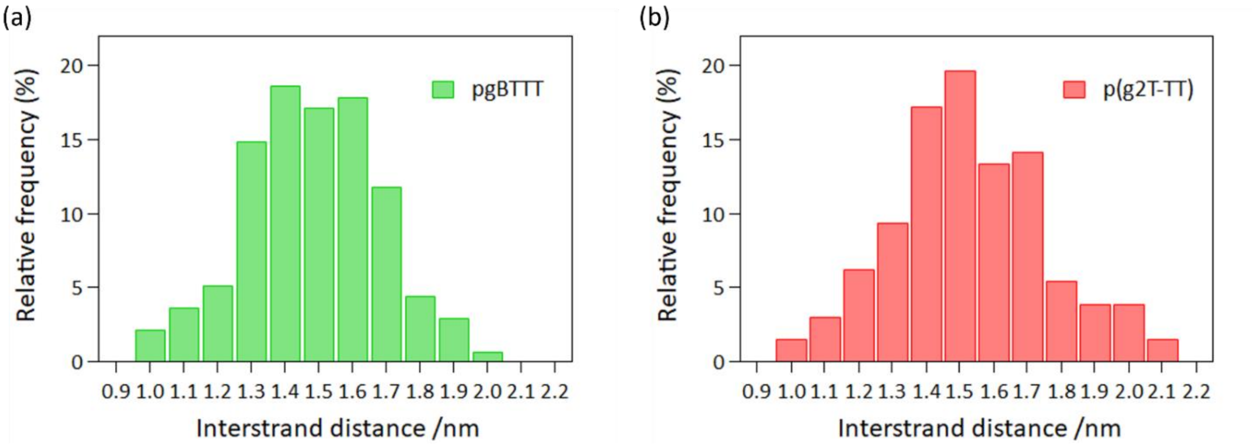

Figure S7. Histograms representing the inter-backbone distance distribution for (a) pgBTTT and (b) p(g2T-TT), measured in high coverage areas.

of the backbones. The collected distances are reported in the histograms of Fig. S6 for pgBTTT and p(g2T-TT), showing a distribution of possible values.

9. Models of interacting glycolated side chains

The helical structure reported by French *et al.*[20] for the crystalline form of PEG_{16} has been used as a model for pairs of interdigitating TEG side chains. Portions of PEG_{16} of appropriate length and always starting with an oxygen atom were isolated by "carving" them out of parallel pairs of PEG_{16} chains in order to create couples of shorter chains that kept the relative distances and conformations of the crystalline form (Figs. S8b-S8g). This was done on the full length of the PEG_{16} chains to extract all possible non-equivalent sections of interacting TEG side chain pairs. Two particular configurations were found displaying a maximum overlap between the TEG chain (Figs. S8e and S78), and these are expected to correspond to the strongest interchain interaction. All other TEG side chain pairs obtained following the

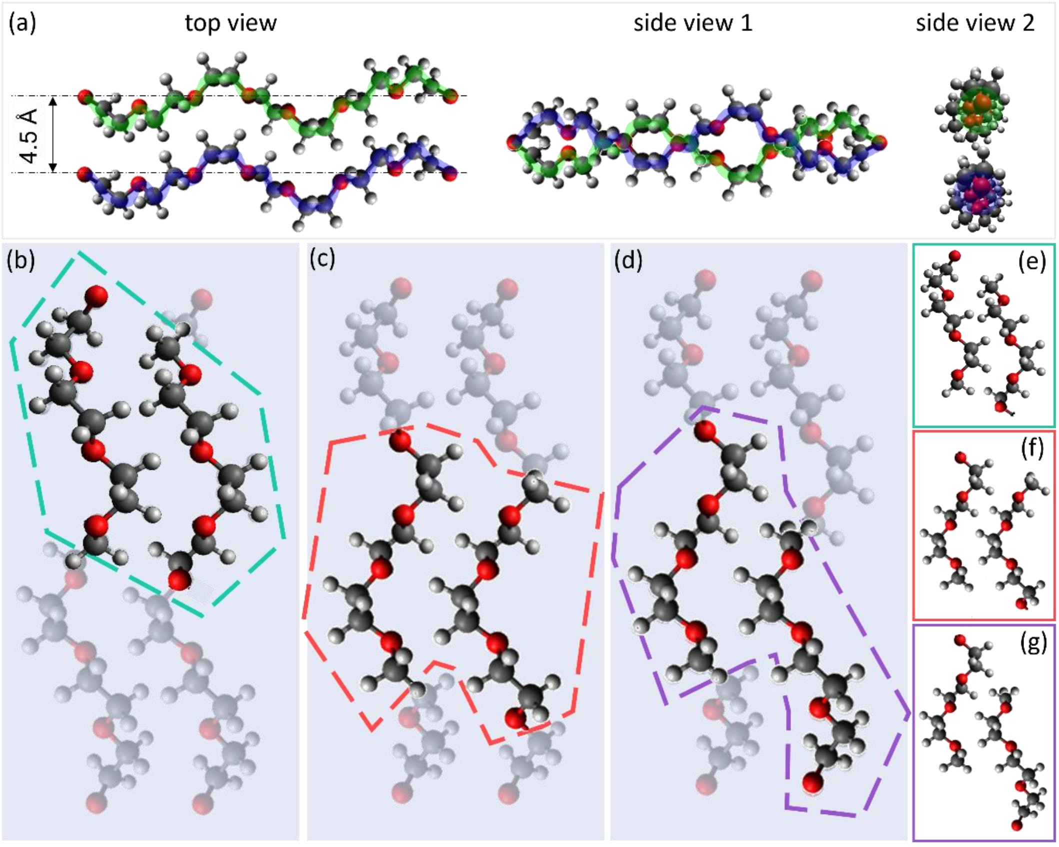

Figure S8. (a) Top and side views of the crystalline structure of PEG_{16} chains as determined by French *et al.*[20]. Only a section of the whole PEG_{16} chains is shown for clarity. (b), (c) and (d) Depictions of the cut-outs resulting in the pairs of TEG side-chains shown in (e), (f) and (g), respectively.

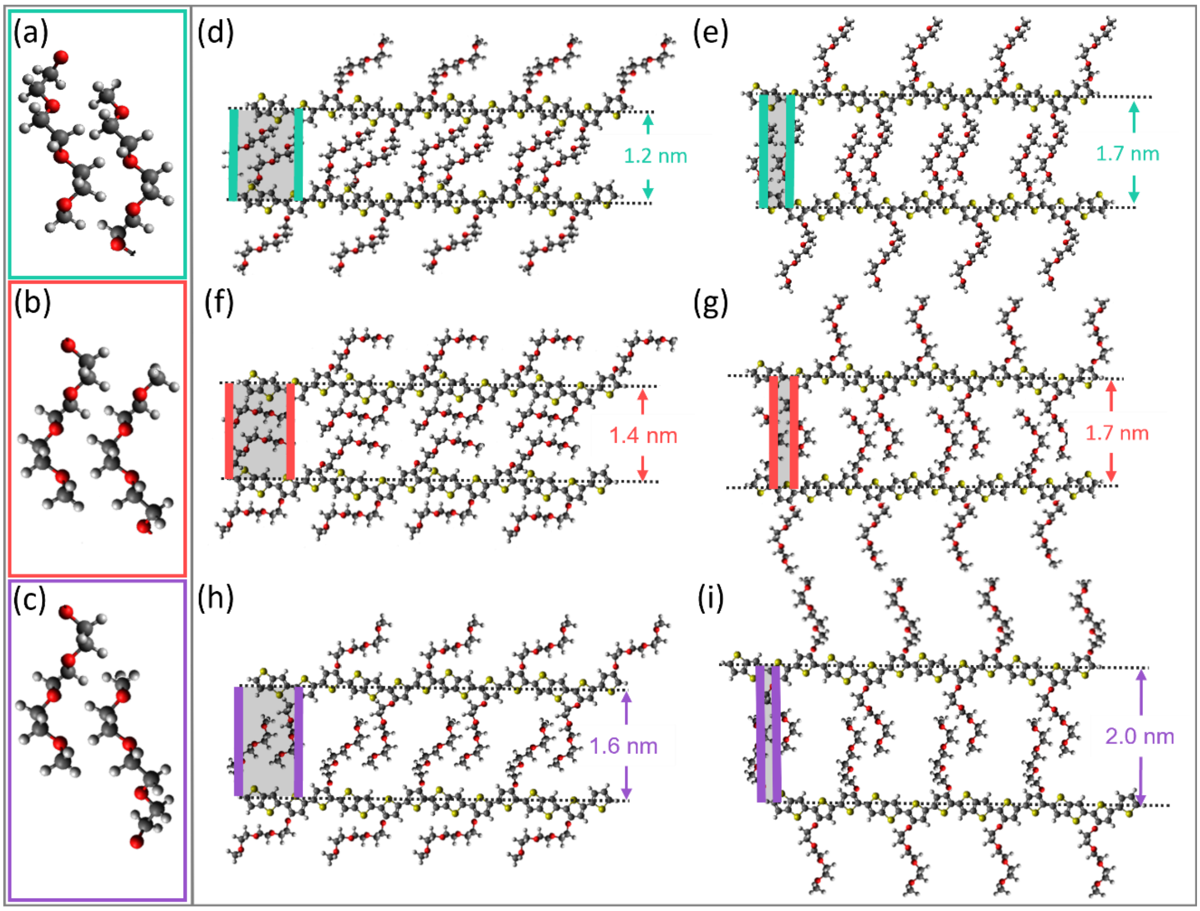

Figure S9. (a-c) Different pairs of interacting TEG chains, "cut-out" from the crystalline form of PEG_{16} (see section 7 of the SI). (d-i) Selected pairs of TEG chains fitted to the 4-4' positions of pgBTTT polymer backbones. The various cases have TEG chains forming different tilt angles with respect to the backbone and display different extents of overlap with those of the neighbouring polymer. The inter-backbone distances obtained from these structures is compatible with the range of values observed experimentally. The parallel coloured lines indicate the distance along the backbones of the attachment positions of the two interacting side-chains (which gives a measure of the registration of the backbones).

described procedure were marked by a lower degree of overlap and thus an expected lower interaction energy (one example is shown in Fig. S8g).

In a second step, selected pairs of TEG chains were used to describe the interaction between polymers by attaching them in their "carved out" conformation onto geometry optimised models of the backbones. Out of all the possible configurations that can be found, in Figs. S9 and S10 (for pgBTTT and p(g2T-TT), respectively) we report the cases that allow to obtain the minimum and the maximum inter-backbone distance. The models in the right column of both figures (e, g, i) are characterised by having the pairs of TEG chains almost perpendicular to the direction of the backbones, resulting in a larger separation between the backbones and a lower local side chain density. The opposite is true for the models in the left column of both

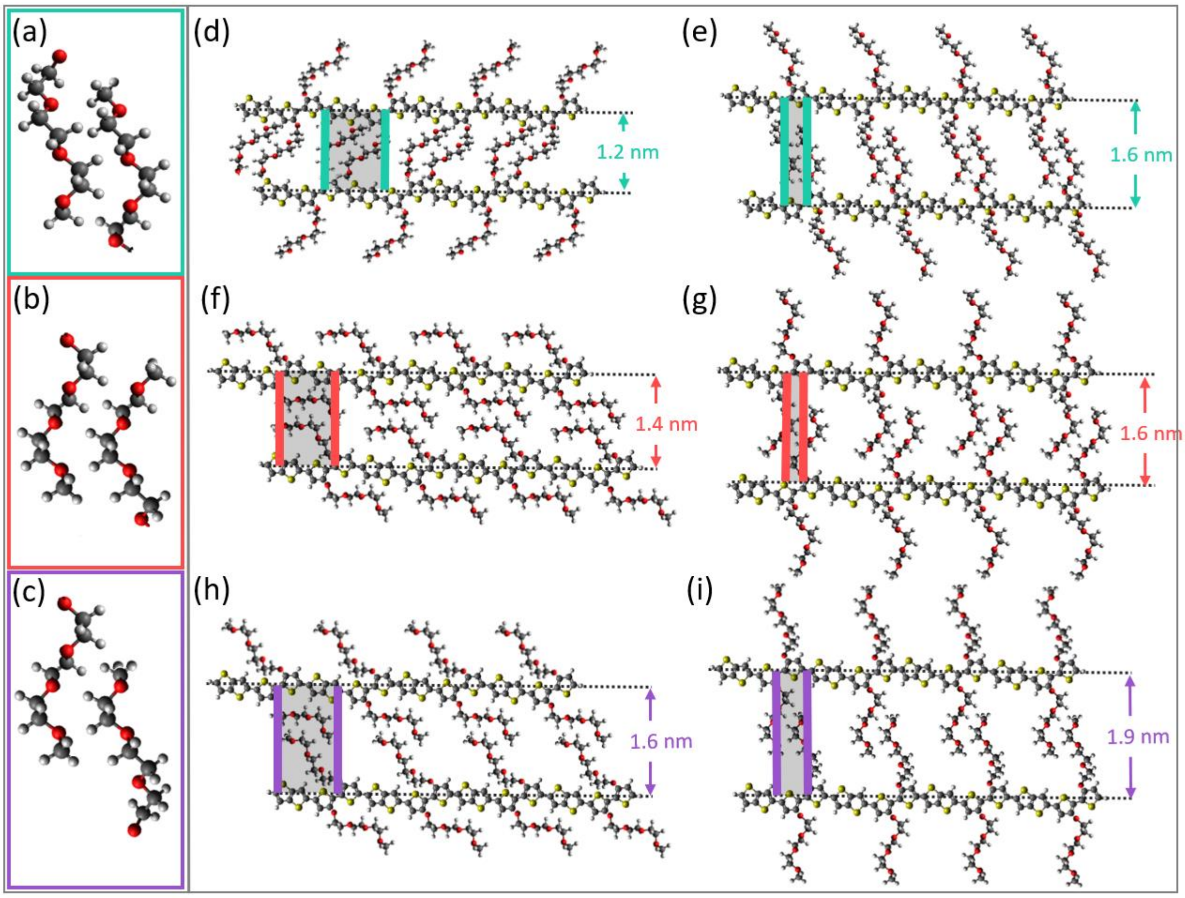

Figure S10. (a-c) Different pairs of interacting TEG chains, "cut-out" from the crystalline form of PEG_{16}. (d-i) Selected pairs of TEG chains fitted to the 3-3' positions of p(g2T-TT) polymer backbones. The various cases have TEG chains forming different tilt angles with respect to the backbone and display different extents of overlap with those of the neighbouring polymer. The inter-backbone distances obtained from these structures is compatible with the range of values observed experimentally. The parallel coloured lines indicate the distance along the backbones of the attachment positions of the two interacting side-chains (which gives a measure of the registration of the backbones).

figures (d, f, h), where the axis of the pairs of TEG chains forms a smaller angle with the direction of the backbones.

10.Fitting of molecular models on STM images for pgBTTT and p(g2T-TT)

In order to prove the validity of the assumptions about the conformation of pairs of interacting TEG side chains discussed in Section 9, high-resolution STM images of both pgBTTT and p(g2T-TT) were fitted with the geometry-optimised molecular models of the polymers displayed in Figs. S9 and S10. In particular, the attachment locations of the side chains along the backbones could be pinpointed on the STM images as the positions of contact of the backbones with the brighter features associated to the side chains (see green dots in Fig. S11c

and red in Fig. S12c). Similarly to the procedure followed in the case of pBTTT, these positions were used to guide the fitting of individual BTTT repeat units (i.e. the backbone's monomer units), taking care that their length and shape was consistent with the experimentally observed "wiggliness" of the backbone (see green lines in Figs. S11c and red ones in Fig. S12c). Once the position of the monomers BTTT was determined within the backbones, a pair of side chains, chosen from those described above, was attached at each exit-point (green and red dots in Figs. S11c and S12c) and rotated until the best match was found with the features displayed in the STM images. Figs. S11d and S12d show the different types of side chains pairs that best matched the STM images, as shown in Figs. 6e and 6f of the main text (reported again as Figs. S11b and S12b, for clarity). In Figs. S11d and S12d, each pair of TEG chains is surrounded by a box, whose colour corresponds to those defined in Figs. S9 and S10. The good agreement of the fit with the STM data allows to satisfactorily identify all features in the images. This procedure can only be successfully attempted in ordered areas of the samples, where high-resolution imaging was possible.

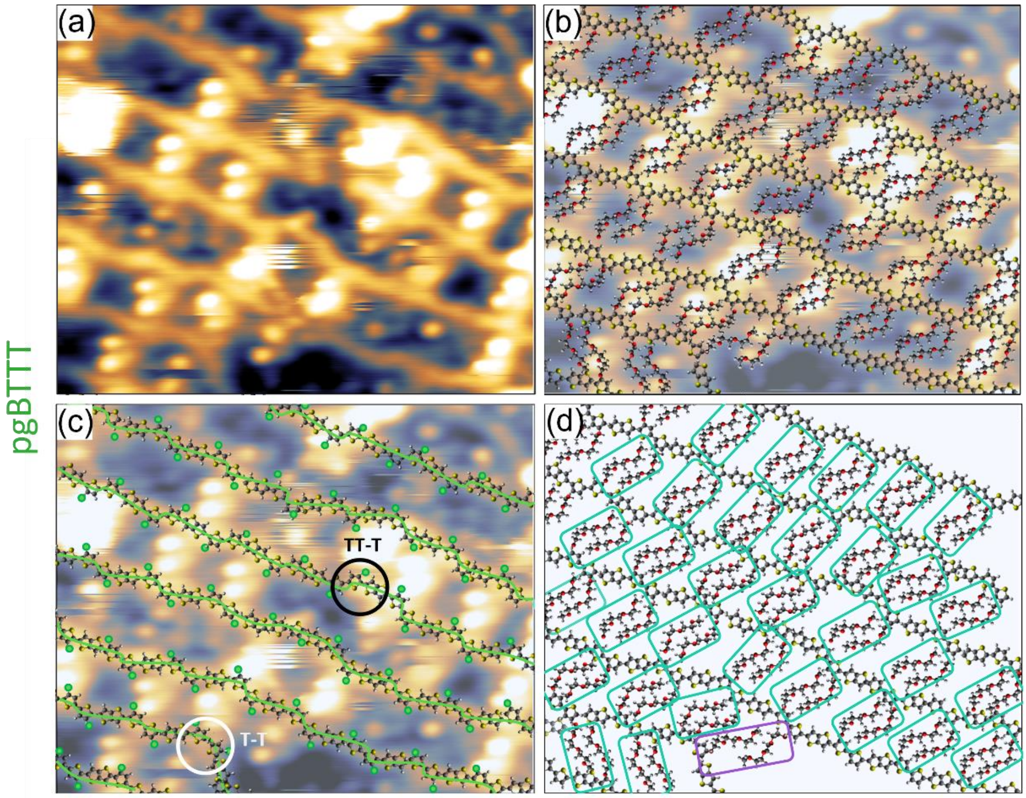

Figure S11. Fitting of molecular models on STM images of pgBTTT. (a) STM image identical to that shown in Fig. 6e of the main text. (b) Final model obtained through the described procedure. (c) Fit of the backbones with single BTTT monomers (indicated also by a green wiggly line), starting from locating the side-chain exit positions (shown as green circles). Examples of *cis* T-T and T-TT bonds are also shown as a white or black circle, respectively. (d) Molecular model with different types of pairs of side-chains indicated by rectangular boxes with colours matching those of Fig. S9.

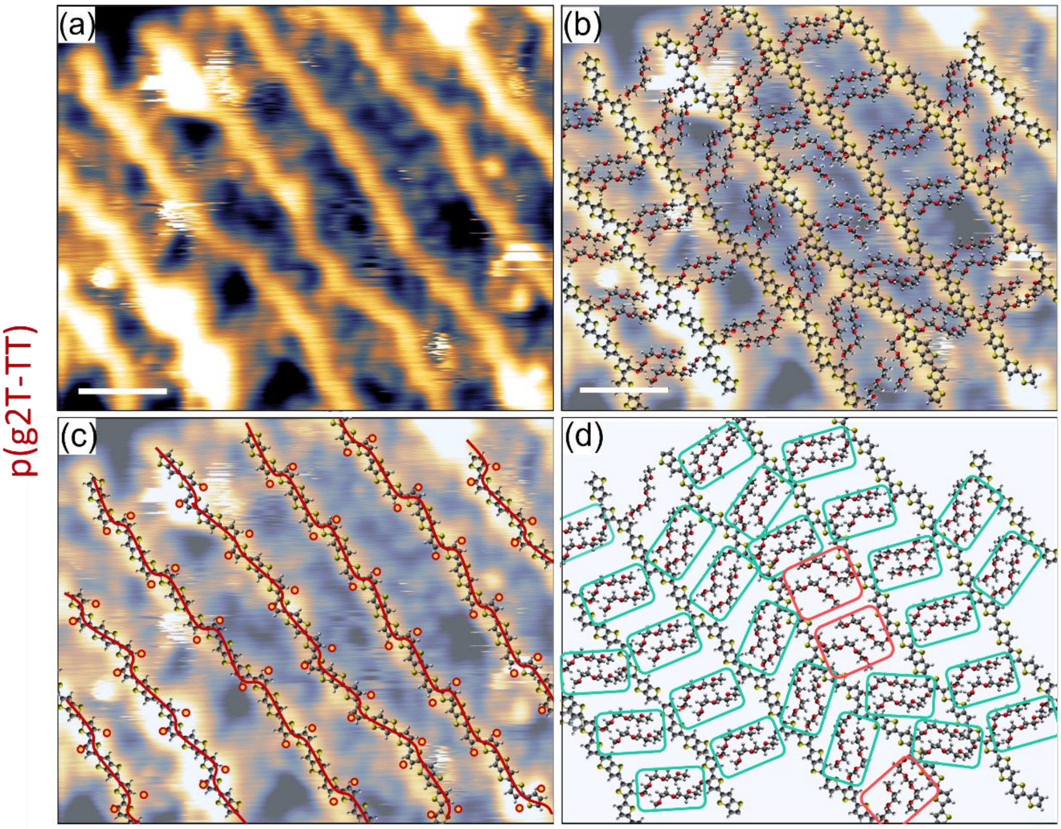

Figure S12. Fitting of molecular models on STM images of p(g2T-TT). (a) STM image identical to that shown in Fig. 6f of the main text. b) Final model obtained through the described procedure. (c) Fit of the backbones with single BTTT monomers (indicated also by a red wiggly line), starting from locating the side-chain exit positions (shown as red circles). In this specific image, there were not any cases of *cis* T-T or T-TT *cis* rotationsbonds. (d) Molecular model with different types of pairs of side-chains indicated by rectangular boxes with colours matching those of Fig. S10.

11. Details of the MD simulations

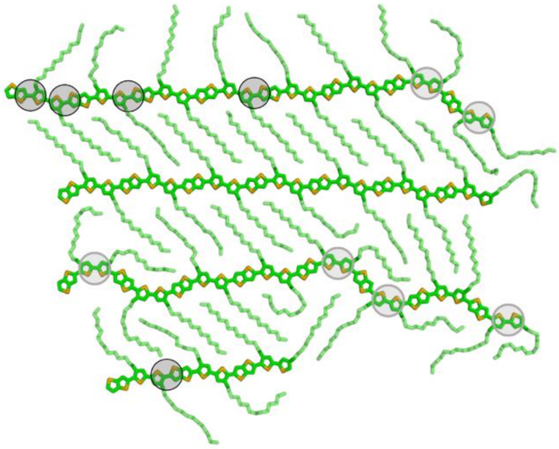

Figure S13. Close-up image of a portion of high density simulated pBTTT-C_{14}. Black circles represent a *cis* T-TT bond, grey circles a *cis* T-T bond. The first do not disturb the general linearity of the backbones, while the latter give rise to pronounced kinks.

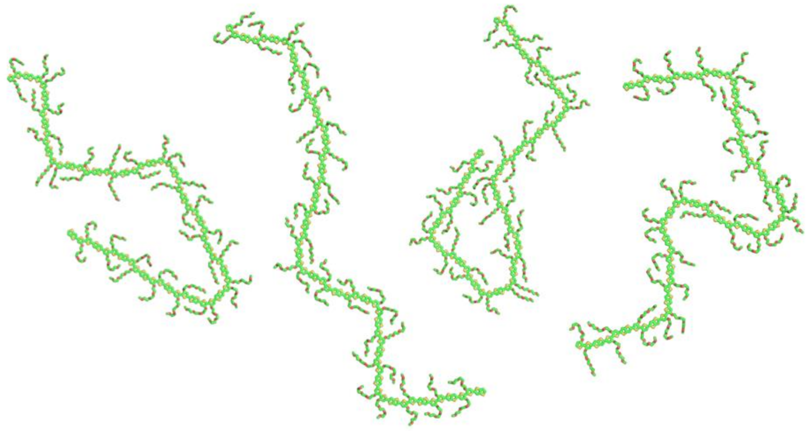

Figure S14. A selection of typical configurations observed when p(g2T-TT) is simulated in the low-density limit. Every simulated replica contains at least one backbone kink, with most containing more. The backbone kinks are clearly identified as being due to a *cis* T-T bond.

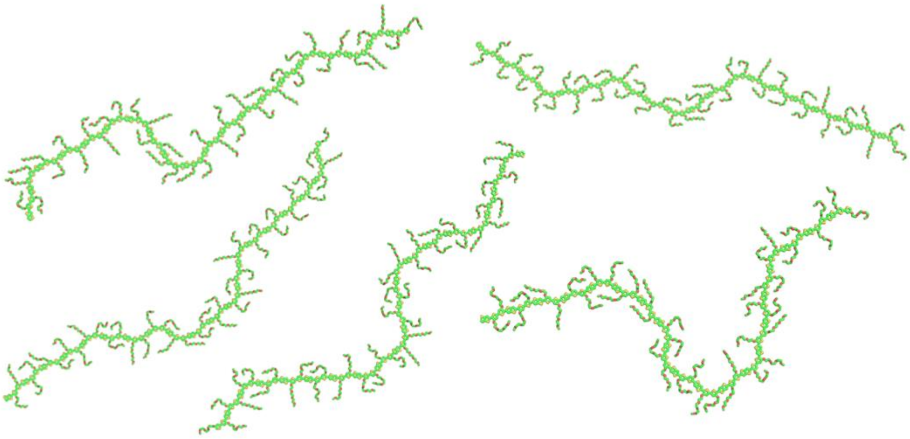

Figure S54. A selection of typical configurations observed when pgBTTT is simulated in the low-density limit. Similar rates of backbone kinking are observed when compared to the case of pg(2T-TT); however, the angles formed by the backbone kinks are less pronounced. This is likely due to the reduced steric hindrance that occurs upon a *cis* T-T bond due to the side chain pairs being positioned further away from each other than in the case of pg(2T-TT).

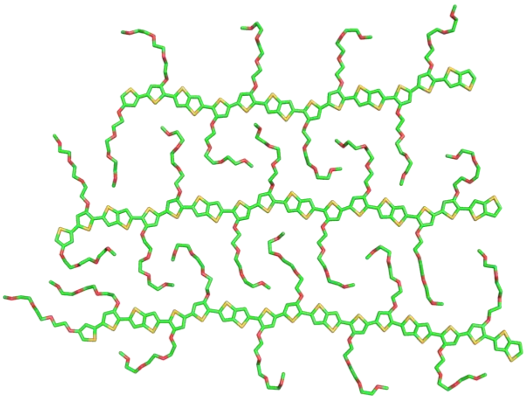

Figure S16. Snapshot from high density MD simulations of pgBTTT showing the side chain pairing seen in MD.

References

1. Horcas, I. *et al.* WSXM: A software for scanning probe microscopy and a tool for nanotechnology. *Rev. Sci. Instrum.* **78**, 013705–013713 (2007).
2. Nečas, D. & Klapetek, P. Gwyddion: An open-source software for SPM data analysis. *Cent. Eur. J. Phys.* **10**, 181–188 (2012).
3. Hanwell, M. D. *et al.* Avogadro: An advanced semantic chemical editor, visualization, and analysis platform. *J. Cheminform.* **4**, 1–17 (2012).
4. LMAPper - The SPM and Mol Viewer | Reviews for LMAPper - The SPM and Mol Viewer at SourceForge.net. https://sourceforge.net/projects/spm-and-mol-viewer/reviews.
5. Jorgensen, W. L., Maxwell, D. S. & Tirado-Rives, J. Development and testing of the OPLS all-atom force field on conformational energetics and properties of organic liquids. *J. Am. Chem. Soc.* **118**, 11225–11236 (1996).
6. Wildman, J., Repiščák, P., Paterson, M. J. & Galbraith, I. General Force-Field Parametrization Scheme for Molecular Dynamics Simulations of Conjugated Materials in Solution. *J. Chem. Theory Comput.* **12**, 3813–3824 (2016).
7. Woods, D. J. *et al.* Side-chain tuning in conjugated polymer photocatalysts for improved hydrogen production from water. *Energy Environ. Sci.* **13**, 1843–1855 (2020).
8. Moreno, M., Casalegno, M., Raos, G., Meille, S. V. & Po, R. Molecular modeling of crystalline alkylthiophene oligomers and polymers. *J. Phys. Chem. B* **114**, 1591–1602 (2010).
9. Bhatta, R. S., Yimer, Y. Y., Perry, D. S. & Tsige, M. Improved force field for molecular modeling of poly(3-hexylthiophene). *J. Phys. Chem. B* **117**, 10035–10045 (2013).

10. Siemons, N. *et al.* Impact of Side Chain Hydrophilicity on Packing, Swelling and Ion Interactions in Oxy-bithiophene Semiconductors. (2022).
11. Pronk, S. *et al.* GROMACS 4.5: A high-throughput and highly parallel open source molecular simulation toolkit. *Bioinformatics* **29**, 845–854 (2013).
12. Van Der Spoel, D. *et al.* GROMACS: Fast, flexible, and free. *J. Comput. Chem.* **26**, 1701–1718 (2005).
13. Allouche, A. Software News and Updates Gabedit — A Graphical User Interface for Computational Chemistry Softwares. *J. Comput. Chem.* **32**, 174–182 (2012).
14. The PyMOL Molecular Graphics System, Version 1.8 Schrödinger, LLC. http://www.sciepub.com/reference/159710.
15. Heinz, H., Vaia, R. A., Farmer, B. L. & Naik, R. R. Accurate simulation of surfaces and interfaces of face-centered cubic metals using 12-6 and 9-6 lennard-jones potentials. *J. Phys. Chem. C* **112**, 17281–17290 (2008).
16. Su, L., Krim, J. & Brenner, D. W. Interdependent Roles of Electrostatics and Surface Functionalization on the Adhesion Strengths of Nanodiamonds to Gold in Aqueous Environments Revealed by Molecular Dynamics Simulations. *J. Phys. Chem. Lett.* **9**, 4396–4400 (2018).
17. Bussi, G., Donadio, D. & Parrinello, M. Canonical sampling through velocity rescaling. *J. Chem. Phys.* **126**, (2007).
18. Parrinello, M. & Rahman, A. Polymorphic transitions in single crystals: A new molecular dynamics method. *J. Appl. Phys.* **52**, 7182–7190 (1981).
19. Nosé, S. & Klein, M. L. Constant pressure molecular dynamics for molecular systems. *Mol. Phys.* **50**, 1055–1076 (1983).
20. French, A. C., Thompson, A. L. & Davis, B. G. High-purity discrete PEG-oligomer crystals allow structural insight. *Angew. Chemie - Int. Ed.* **48**, 1248–1252 (2009).
